# An Integrated Meta-Analysis of Secretome and Proteome Identify Potential Biomarkers of Pancreatic Ductal Adenocarcinoma

**DOI:** 10.3390/cancers12030716

**Published:** 2020-03-18

**Authors:** Grasieli de Oliveira, Paula Paccielli Freire, Sarah Santiloni Cury, Diogo de Moraes, Jakeline Santos Oliveira, Maeli Dal-Pai-Silva, Patrícia Pintor do Reis, Robson Francisco Carvalho

**Affiliations:** 1 Department of Structural and Functional Biology, Institute of Biosciences, São Paulo State University (UNESP), Botucatu 18618-689, São Paulo, Brazil; grasieli.oliveira@unesp.br (G.d.O.); paula.freire@unesp.br (P.P.F.); sarahscury@gmail.com (S.S.C.); demoraesdiogo2017@gmail.com (D.d.M.); jakoliveira.jo@gmail.com (J.S.O.); maeli.dal-pai@unesp.br (M.D.-P.-S.); 2 Department of Surgery and Orthopedics, Faculty of Medicine, São Paulo State University (UNESP), Botucatu 18618-687, São Paulo, Brazil; patricia.reis@unesp.br; 3 Experimental Research Unity, Faculty of Medicine, São Paulo State University, UNESP, Botucatu 18618-970, São Paulo, Brazil

**Keywords:** PDAC, mass spectrometry, bioinformatics, prognostic biomarkers

## Abstract

Pancreatic ductal adenocarcinoma (PDAC) is extremely aggressive, has an unfavorable prognosis, and there are no biomarkers for early detection of the disease or identification of individuals at high risk for morbidity or mortality. The cellular and molecular complexity of PDAC leads to inconsistences in clinical validations of many proteins that have been evaluated as prognostic biomarkers of the disease. The tumor secretome, a potential source of biomarkers in PDAC, plays a crucial role in cell proliferation and metastasis, as well as in resistance to treatments, which together contribute to a worse clinical outcome. The massive amount of proteomic data from pancreatic cancer that has been generated from previous studies can be integrated and explored to uncover secreted proteins relevant to the diagnosis and prognosis of the disease. The present study aimed to perform an integrated meta-analysis of PDAC proteome and secretome public data to identify potential biomarkers of the disease. Our meta-analysis combined mass spectrometry data obtained from two systematic reviews of the pancreatic cancer literature, which independently selected 20 studies of the secretome and 35 of the proteome. Next, we predicted the secreted proteins using seven in silico tools or databases, which identified 39 secreted proteins shared between the secretome and proteome data. Notably, the expression of 31 genes of these secretome-related proteins was upregulated in PDAC samples from The Cancer Genome Atlas (TCGA) when compared to control samples from TCGA and The Genotype-Tissue Expression (GTEx). The prognostic value of these 39 secreted proteins in predicting survival outcome was confirmed using gene expression data from four PDAC datasets (validation set). The gene expression of these secreted proteins was able to distinguish high- and low-survival patients in nine additional tumor types from TCGA, demonstrating that deregulation of these secreted proteins may also contribute to the prognosis in multiple cancers types. Finally, we compared the prognostic value of the identified secreted proteins in PDAC biomarkers studies from the literature. This analysis revealed that our gene signature performed equally well or better than the signatures from these previous studies. In conclusion, our integrated meta-analysis of PDAC proteome and secretome identified 39 secreted proteins as potential biomarkers, and the tumor gene expression profile of these proteins in patients with PDAC is associated with worse overall survival.

## 1. Introduction

Pancreatic cancer includes both exocrine and endocrine tumors, of which the majority are pancreatic ductal adenocarcinomas (PDACs)—one of the most aggressive and lethal human cancers among solid malignancies [1]. Due to its poor prognosis, with the number of deaths (*n* = 432,000) nearly equivalent to the number of cases (*n* = 459,000), pancreatic cancer is the seventh leading cause of cancer death [2]. Less than 5% of patients survive up to 5 years after diagnosis [3,4]. Because of the asymptomatic nature of the early stages of the disease and the lack of efficient methods for its detection, most patients with pancreatic cancer have locally advanced and inoperable disease at diagnosis [5,6]. Also, about 90% of the PDAC cases are complex due to distant metastases [7,8]. The disease becomes even more complicated in the advanced stages because chemotherapy, radiotherapy, and combined therapies become severely impaired [3,9,10]. Less than 20% of patients are candidates for complete surgical removal, and even after treatment, most relapse and die within a year [11].

The lack of significant progress in clinical treatment compared to other cancer types is attributable to the current inability to develop new and effective therapies; current standard treatments still consist of surgical resection and cytotoxic therapies [12]. Therapeutic options are limited, and the progress in drug development is hindered because most PDACs are highly complex at the cellular, genomic, epigenomic, and metabolic levels [13,14]. Furthermore, the interaction between neoplastic and stromal cells in tumor microenvironment challenges medical treatment [15]. Current alternatives to study the molecular complexity of PDAC lies in the integration of multi-omics profiles and experimental meta-data, which might lead to the identification of new drugs and therapeutic targets, as well as the discovery of biomarkers to improve diagnostic, prognosis, chemotherapy responses, and prediction of tumor recurrence [16].

Several studies have identified gene expression signatures using tumor-derived transcriptome data from PDAC patients, targeting the development of better prognostic tools able to stratify patient survival, relapse, and treatment response [17,18,19,20,21,22,23,24]. Although these gene signatures predict patient survival; the implementation of biomarkers in clinical practice has not yet been possible, in part because many of these signatures were derived from gene expression data that may not accurately reflect changes in plasma proteins [24,25,26]. Advances in RNA and DNA sequencing technologies that enable large-scale analysis of the molecular characteristics from histopathologically indistinguishable tumors have often shown substantial molecular differences that preclude the guidance of a significant amount of clinical decisions [13]. This PDAC cellular and molecular heterogeneity culminate in an immense dynamic range of protein quantity coupled with a plethora of isoforms that also challenge the discovery of protein-based biomarkers [27].

Protein-based biomarkers have the potential to bring benefits to clinical predictions of the disease [28]. Recent advances in cancer proteomics have paralleled the development of different proteomics approaches and technologies [27], increasing the number of pancreatic cancer-related proteins that have been identified in tissues, body fluids (such as pancreatic juice, plasma, and serum), and in cancer cell lines [29,30,31]. The action of soluble secreted mediators (secretome) by cancer cells and cells within the tumor microenvironment has been recognized as one of the main factors influencing tumor biology [32]. The secretion of functional biomolecule-enriched exosomes not only allows pancreatic cancer cells to model the activity of adjacent cells in their tumor microenvironment but also enables them to exploit distant cells to optimize conditions for future metastatic seeding [33]. PDAC-derived exosomes expressing the Macrophage Migration Inhibitory Factor (MIF) induce the formation of a pre-metastatic niche in the mouse liver, and therefore this protein can be used as a prognostic marker for the development of liver metastasis [34]. Glypican 1 (GPC1) expression in blood exosomes of pancreatic cancer patients was able to distinguish patients with benign or malign disease with absolute specificity and sensitivity [35]. Therefore, this protein also has a noninvasive diagnosis potential to detect the early stages of PDAC. These findings provide interesting information on how tumor cells can communicate and promote tumor progression through oncogenic protein transfer [36]. Thus, focusing on proteomic analyses of the cancer secretome is an exciting approach once it represents the portion of the proteome expected to harbor promising biomarkers [37].

Proteomics analyses of PDAC have identified a vast amount of cancer-associated proteins [32,38,39,40,41,42,43,44,45,46], including secreted proteins [47]. Recent evidence supports the concept that protein biomarker signatures, as opposed to individual biomarkers, may ensure the long-sought accuracy in cancer diagnosis [48]. Thus, we used publicly available data to perform an integrative meta-analysis of pancreatic cancer tumor cell secretome and tumor proteome to identify potential biomarkers of the disease. This discovery set allowed us to identify 39 proteins that were confirmed as secreted by prediction analyses using seven in silico tools or databases. We also compared the transcriptomic profiles from PDAC and normal tissue samples using The Cancer Genome Atlas (TCGA) and The Genotype-Tissue Expression (GTEx) data, which revealed that 31 secreted genes are upregulated in PDAC. Next, we determined the prognostic value of the 31 secreted proteins for predicting survival outcome using gene expression data from four PDAC datasets (validation set) and in nine additional tumor types from TCGA. In all these datasets, the potentially secreted proteins were capable of distinguishing better and worse prognosis (overall survival). Finally, we compared the prognostic value of our set with sets of other PDAC biomarkers studies. Our gene signature performed equally well or better than the signatures found in previous studies in terms of its ability to predict prognosis, but it is the only one based on genes that are translated into secreted proteins and, therefore, can be potentially used as plasma or tumor microenvironment biomarkers. In conclusion, the integration of proteome and secretome mass spectrometry data from pancreatic cancer tumors and cells identified 39 secreted proteins, and tumor gene expression profile of these proteins in patients with PDAC is associated with worse overall survival. This list of protein-coding genes in PDAC may serve as potential biomarkers or as targets for new therapeutic approaches of the disease.

## 2. Results

### 2.1. Integration of Secretome and Proteome Meta-Analysis Identifies 39 Secreted Proteins in Pancreatic Ductal Adenocarcinoma

**Figure 1:**
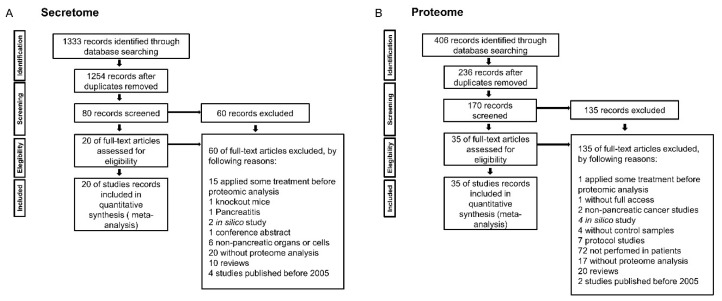
Flowchart of the integrative secretome (**A**) and proteome (**B**) meta-analyses in pancreatic cancer.

**Figure 2:**
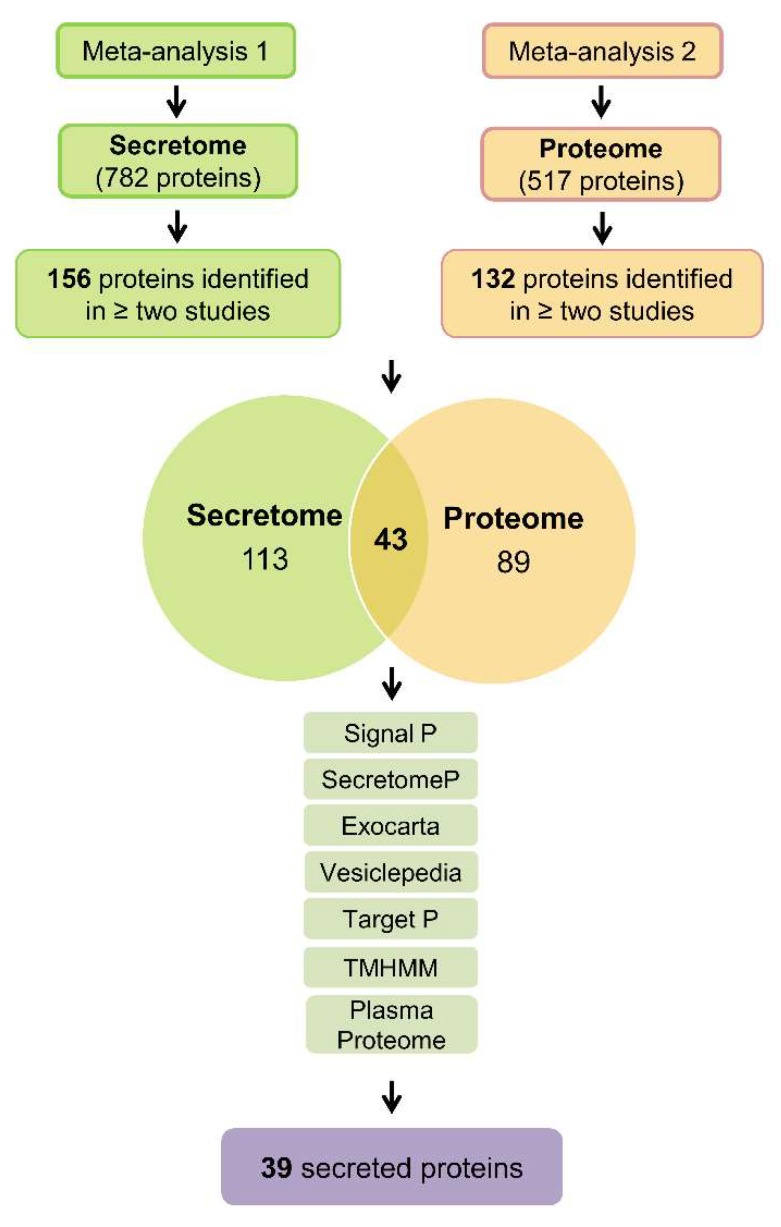
Schematic representation of the workflow used to identify secreted proteins in pancreatic cancer studies. Two meta-analyses of publicly available proteomic studies were used to identify secreted proteins in pancreatic ductal adenocarcinomas: meta-analysis 1 identified 782 proteins in the secretome, and meta-analysis 2 identified 517 proteins in the proteome. Subsequently, the proteins present in two or more studies were selected. This strategy resulted in a final list containing 156 proteins in the secretome (meta-analysis 1) and 132 proteins in the proteome (meta-analysis 2). The intersection between the proteins identified 43 shared proteins between the two meta-analyses. These 43 proteins were further verified as secreted using the algorithms available at Center for Biological Sequencing Analysis (CBS): SignalP [94] (identifies classical secreted proteins, presence of signal peptide); SecretomeP [95] (identifies non-classical secreted proteins); and the databases Vesiclepedia [96] (protein data in secretory vesicles), ExoCarta [97] (protein data in exosomes) and Plasma Proteome [98] (protein data identified in the blood). Proteins were excluded from further analysis if detected by the CBS TargetP [99] (mitochondrial protein) or TMHMM [100] (transmembrane helix protein) algorithms, resulting in a final list of 39 proteins in the pancreatic ductal adenocarcinoma shared by proteome and secretome studies.

We performed an integrative meta-analysis on pancreatic cancer secretome and proteome data to identify clinically relevant diagnostic and prognostic biomarkers. According to the meta-analysis study design and inclusion and exclusion criteria (Figure 1), 20 secretome studies and 35 proteome studies in pancreatic cancer were selected, which reported protein data obtained by mass spectrometry (Table 1 and Table 2) [3,6,29,34,37,41,43,45,46,47,49,50,51,52,53,54,55,56,57,58,59,60,61,62,63,64,65,66,67,68,69,70,71,72,73,74,75,76,77,78,79,80,81,82,83,84,85,86,87,88,89,90,91,92,93]. These data identified 782 proteins in pancreatic cancer secretome and 517 proteins in pancreatic ductal adenocarcinoma tumor samples. Interestingly, we did not identify a protein shared by all secretome and proteome studies. Therefore, we chose to select proteins present in two or more studies. This approach allowed us to obtain 156 and 132 proteins in the pancreatic cancer secretome and proteome, respectively (Figure 2 and Tables S1–S4). The intersection between the secretome and proteome protein lists revealed 43 proteins in common between the two meta-analysis strategies (Figure 2). These 43 shared proteins were further verified as secreted using prediction analysis regarding the nature of secretion (Figure 2). These analyses confirmed the selection of proteins that are not be derived from other mechanisms such as cell death. Among the 43 proteins verified by SignalP, SecretomeP, Exocarta, Vesiclepedia, TargetP, and TMHMM, the proteins Vimentin (VIM), Glyceraldehyde-3-phosphate dehydrogenase (GAPDH), and Superoxide dismutase 2 (SOD2) were predicted to contain mitochondrial sublocation (TargetP) and the Transforming growth factor beta induced (TGFBI) protein was predicted to contain transmembrane helices (TMHMM). These four proteins were eliminated, resulting in a final list of 39 proteins predicted as secreted. This set of proteins is also detected in plasma, as confirmed in the Plasma Proteome database (Table 3 and Table S5).

### 2.2. Protein-Protein Interaction (PPI) Network of 39 Secreted Proteins Enriched in Pancreatic Ductal Adenocarcinoma

**Figure 3:**
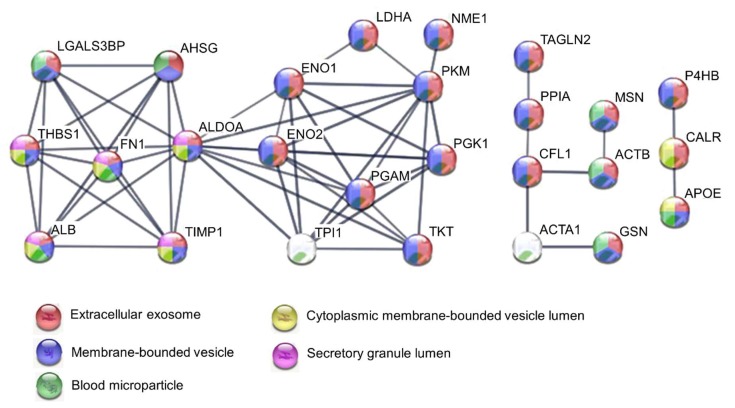
Protein-protein interaction network of 39 secreted proteins enriched in pancreatic ductal adenocarcinoma. Analysis using STRING [101] illustrates potential interactions between secreted proteins with a minimum confidence score of 0.7. Proteins in the interaction network are represented as nodes connected by lines whose thickness reflects a confidence index higher than 0.7. The top five enriched GO terms are represented by the network node colors for each protein: red, extracellular exosome; blue, membrane-bounded vesicle; green, blood microparticle; yellow, cytoplasmic membrane-bounded vesicle lumen; purple, secretory granule lumen.

The PPI network and gene ontology (GO) of the 39 proteins were generated using the Search Tool for Retrieval of Interacting Genes (STRING) database [101]. Data from this database revealed a complex interaction network between 26 proteins with strong associations represented by thick lines; the disconnected nodes in the network were hidden (Figure 3). Gene Ontology analysis revealed significant protein enrichment in categories of extracellular exosomes, membrane-bound vesicles, extracellular part, and blood microparticles (Figure 3 and Table 4). This analysis also showed involvement in biological processes such as glycolysis, regulation of apoptotic processes, vesicle-mediated transport, and stress response. The enriched molecular function was enzymatic and RNA binding (Table 4). These results further confirm our findings showing that the proteins identified in our integrative pancreatic cancer secretome meta-analysis are secreted and located mainly in extracellular compartments.

### 2.3. Secretome-Related Gene Expression is Enriched in Pancreatic Ductal Adenocarcinoma

**Figure 4:**
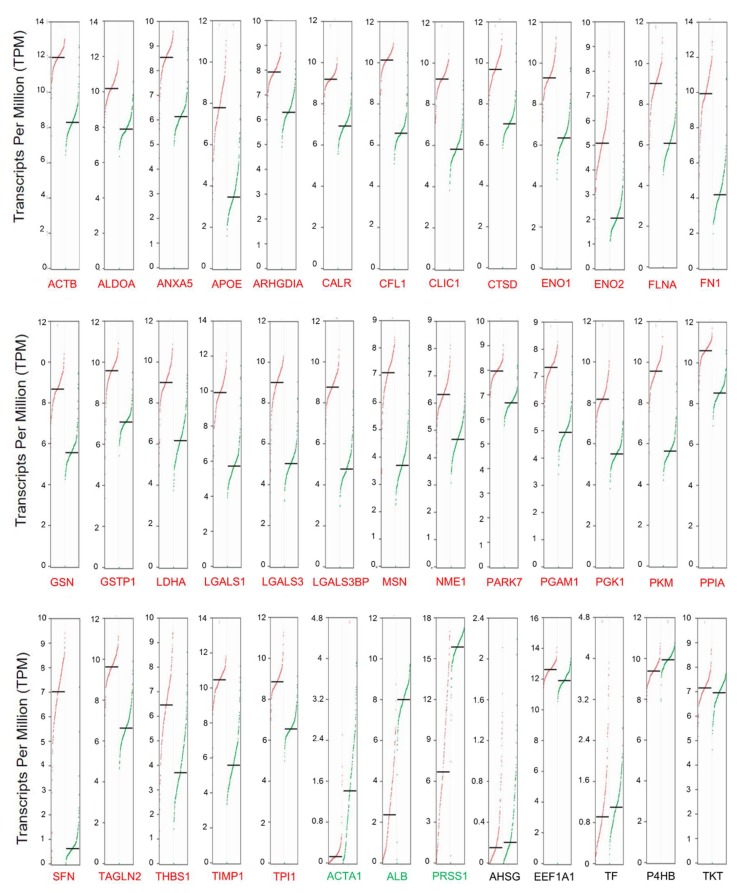
Differential gene expression of transcripts translated into secreted proteins in pancreatic ductal adenocarcinoma. Differential expression levels were calculated using the web-based Gene Expression Profiling Analysis tool (GEPIA) [102]. GEPIA analysis revealed that most of the 39 genes of the proteins identified as secreted are positively regulated in pancreatic ductal adenocarcinoma (PDAC). Genes were considered positively or negatively regulated (written in red and green, respectively) in PDAC (*n* = 179) relative to normal tissue (TCGA, *n* = 4; GTEx, *n* = 167) when absolute values of fold-change were >1.0 and the *q*-value < 0.01 (ANOVA). Red dots: pancreatic tumor samples; Green dots: normal pancreatic samples.

**Figure 5:**
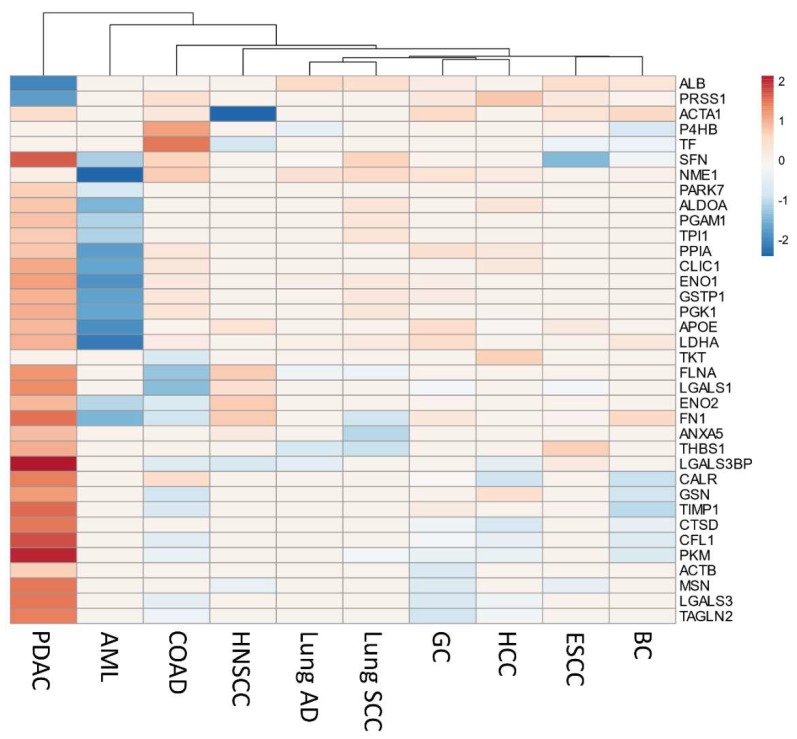
The upregulation of secretome genes is strongly associated with pancreatic ductal adenocarcinoma (PDAC). Heatmap of expression levels of genes [log2 (TPM + 1)] encoding 39 secreted proteins identified as secreted in PDAC, in 10 different tumor types from TCGA compared to their respective normal tissues. Genes that are specifically upregulated or downregulated genes in each tumor type (absolute values of fold-change > 1.0 and *q*-value < 0.01; ANOVA) are shown in red and blue, respectively. Both columns (tumor types) and rows (secretome genes) were clustered using Euclidian distance. Three genes (*ARHGDIA*, *AHSG*, and *EEF1A1*) presented missing values in 90%–100% of the tumor types and are not represented in the heatmap (constant rows were removed during data pre-processing). The differential expression levels from tumor tissue versus combined normal TCGA and GTEx data were calculated using the web-based Gene Expression Profiling Analysis tool (GEPIA) [102]. GC: Gastric carcinoma; COAD: Colon adenocarcinoma; HCC: Hepatocellular carcinoma; Lung SCC: Lung squamous cell carcinoma; BC: Breast carcinoma; HNSCC: Head and neck squamous cell carcinoma; ESCC: Esophageal squamous cell carcinoma; Lung AD: Lung adenocarcinoma; AML: Acute myeloid leukemia.

The gene expression levels of the secretome proteins identified in our integrative meta-analysis were analyzed using the online Gene Expression web-based Profiling Analysis (GEPIA) tool [102]. This tool allows the comparison of transcriptome profiles from TCGA and GTEx using uniformly processed and unified RNA sequencing data by Toil Pipeline [103]. We obtained gene expression profiles of 4743 tumors comprising 10 cancers (TCGA) and 2737 corresponding normal tissues (TCGA and GTEx) (Table S6). The tumors types: gastric carcinoma (GC), colon adenocarcinoma (COAD), Hepatocellular carcinoma (HCC), Lung squamous cell carcinoma (Lung SCC), Breast carcinoma (BC), Head and neck squamous cell carcinoma (HNSCC), Esophageal carcinoma (ESCC), Lung adenocarcinoma (Lung AD), and Acute myeloid leukemia (AML) were selected to allow a comparison between some prevalent cancers. The expression profile of the 39 genes encoding secreted proteins was analyzed using the GEPIA tool to identify prognostic biomarkers of pancreatic ductal adenocarcinoma. The analysis revealed that 31 genes are significantly upregulated in PDAC (Log2 fold change > one and *q*-values cutoff = 0.01) when compared to normal tissues (Figure 4). When we extended the analysis to other TCGA cancers types, the gene expression profile of the 39 secreted-related proteins genes was enriched in PDAC, despite sharing a high number of significantly upregulated genes with other cancers types such as GC, COAD, and HCC (Figure 5, Figure S1A). In this analysis, we can also observe that *ARHGDIA* and *PARK7* are exclusively upregulated in PDAC (Figure S1A). The Euclidean cluster analysis performed on the 39 secretome genes demonstrates that PDAC exhibits a gene expression profile that is clearly distinct from other cancers (Figure 5). This analysis also demonstrated the opposite expression profile for acute myeloid leukemia (AML) (Figure 5). The principal component analysis (PCA) of the expression profiles of the 39 secretome genes in 10 tumor types showed that PDAC and AML are distinguished between cancer types based on the gene expression profile of the 39 secretome proteins and are capable of clearly distinguish PDAC from other cancer types (Figure S2).

We also evaluated the expression profile of the secretome proteins in normal tissues in order to find pancreas-specific proteins deregulated in PDAC. With the help of the GTEx database [104], the expression profiles of the majority of the proteins identified in our study are uniformly expressed across the tissues analyzed (Figure S1B); however, trypsin-1 (*PRSS1*) is upregulated explicitly in normal pancreatic tissue when compared to other normal tissues (Figure S1B). Interestingly, we found that PRSS1 is negatively expressed in PDAC (Figure 4 and 5), although it has often been related to pancreatic cancer [105,106].

### 2.4. Secretome-Related Gene Expression Profile of 39 Proteins Predict Shorter Survival in Patients with Pancreatic Ductal Adenocarcinoma

**Figure 6:**
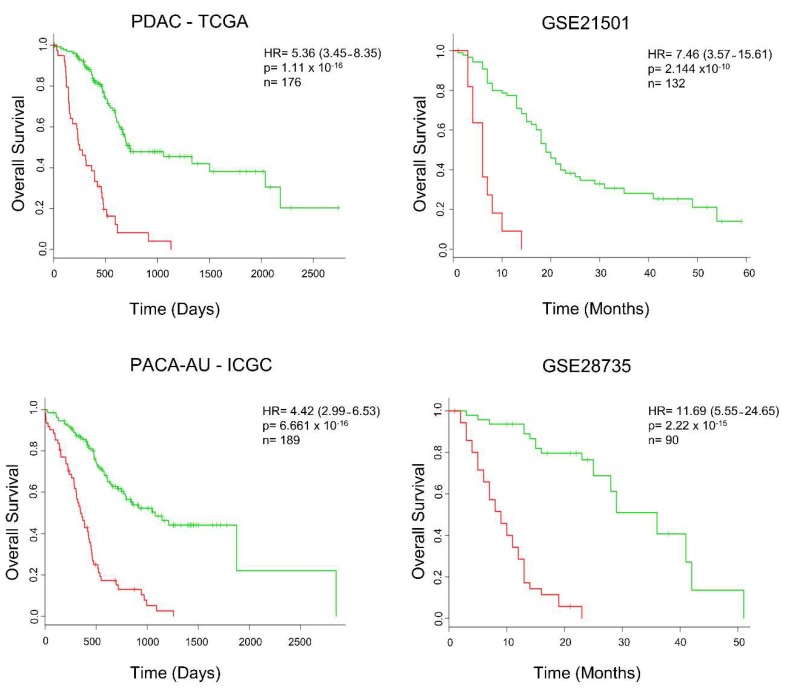
The expression of secretome genes predicts cancer outcomes across four pancreatic ductal adenocarcinoma (PDAC) studies. Survival analysis, based on the expression of 39 mRNAs translated into proteins identified as secreted in PDAC, was calculated using the online platform SurvExpress [107], in four additional and independent PDAC datasets. Cancer patients were stratified into high- (red) and low-risk (green) groups. The adjusted risk ratio (HR) with the corresponding 95% confidence interval, log-rank *p*-value (P), and the number of successfully stratified patients (N) determined by Cox univariate regression analysis is shown in each Kaplan–Meier survival plot. Datasets of PDAC patients: PDAC-TCGA [108], PACA-AU—ICGC [109], GSE21501 [19], and GSE28735 [20,79].

Secretome components from the tumor environment, including proinflammatory cytokines, play a fundamental role in the development of alterations that result in proliferation, metastasis, and resistance to treatments [32,36,44]. Considering that these alterations contribute to the worse prognosis of patients with PDAC, we determine whether the levels of tumor expression of the 39 secretome genes correlate with the patient’s prognosis. We used the SurvExpress platform [107], a web-based biomarker validation tool that provides survival analysis and risk assessment of cancer datasets, to assess whether our set of secretome proteins was able to discriminate overall survival in patients with PDAC. SurvExpress generated a prognostic index (risk score) based on gene expression of the 39 secretome proteins and survival data of the patients with PDAC. These patients were divided into two groups, high- and low-risk, maximizing the number of patients in risk groups by employing an ordered prognostic index optimization algorithm in SurvExpress (Table S7). We evaluated the prognostic value of the gene expression of the 39 secreted proteins and analyzed their association with survival of patients with PDAC (Cox regression analysis) in four different data sets with patient survival information (TCGA, PACA-AU—ICGC, GSE21501, GSE28735). The 39 secretome genes initially analyzed in the TCGA dataset identified patients with significantly shorter survival (hazard ratio (HR) = 5.36; log-rank *p*-value = 1.16–16; concordance index (CI) = 74.75; *n* = 176). In order to validate our findings, survival analysis was performed on three additional independent data sets. These 39 secretome genes were also significantly associated with patient´s outcome in the ICGC datasets (HR = 4.42; CI = 72.57, *p* =6.661–16, *n* = 189), GSE21501 (HR = 7.46; CI= 76.68; *p* = 2.144–10; *n* = 132), and GSE28735 (HR = 11.69; CI= 84.52; *p* = 2.22–15; *n* = 90) (Figure 6). This analysis also demonstrated increased expression of *LDHA*, *ENO1*, and *PGK1* in the TCGA, PACA-AU—ICGC, and GSE21501 datasets; *NME1* was upregulated in all PDAC datasets (Table S8). Enrichment of secretome genes in PDAC patients with low survival (high-risk group) can be observed in the heatmap generated by cluster gene expression analysis (Figure S3). This shows the robustness of the gene expression signature of our set of secreted proteins, which demonstrated significant association with patient survival in the independent PDAC validation sets.

### 2.5. Secretome Gene Expression Predicts Cancer Outcomes in Different Cancer Studies

**Figure 7:**
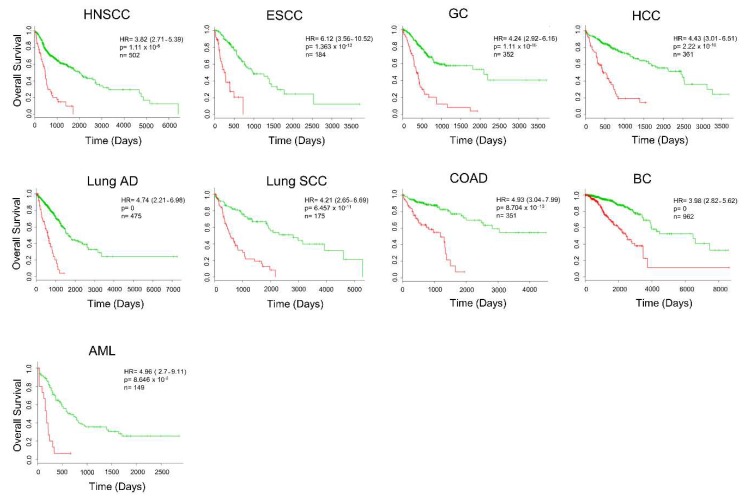
The expression of secretome genes predicts cancer outcomes. Survival analysis based on the expression of 39 transcripts translated into secreted proteins by using the online platform SurvExpress [107] in nine The Cancer Genome Atlas (TCGA) studies. Cancer patients were stratified into high- (red) and low-risk (green) groups. The adjusted risk ratio (HR) with the corresponding 95% confidence interval, log-rank *p*-value (P), and the number of successfully stratified patients (N) determined by Cox univariate regression analysis is shown in each Kaplan–Meier survival plot. HNSCC: Head and neck squamous cell carcinoma; ESCC: Esophageal squamous cell carcinoma; GC: Gastric carcinoma; HCC: Hepatocellular carcinoma; Lung AD: Lung adenocarcinoma; Lung SCC: Lung squamous cell carcinoma; COAD: Colon adenocarcinoma; BC: Breast carcinoma; AML: Acute myeloid leukemia.

Considering that several of our 39 secretome genes are upregulated in other cancer types, we decided to evaluate the prognostic value of the genes in the additional nine different malignancies from TCGA. We found that, in addition to PDAC, our set of 39 genes of secretome-related proteins predicts a worse prognosis in HNSCC, ESCC, GC, HCC, Lung SCC, Lung AD, COAD, BC, and AML (Figure 7 and Table S9). The heatmap for each of these cancer types shows that the expression profile of our set of 39 secretome-related proteins is able to distinguish patients into high- and low-risk groups (Figure S3). The robustness of our 39 secretome genes in stratifying patients with high confidence in risk-groups was confirmed by high hazard ratios in nine TCGA cohorts, showing that changes in the expression of these 39 secretome genes are associated with worse overall survival (Figure 7). Therefore, these results suggest that the set of these 39 secretome genes are predictors of cancer survival outcomes.

### 2.6. Comparison with Prognostic Gene Signatures of Pancreatic Ductal Adenocarcinoma

**Figure 8:**
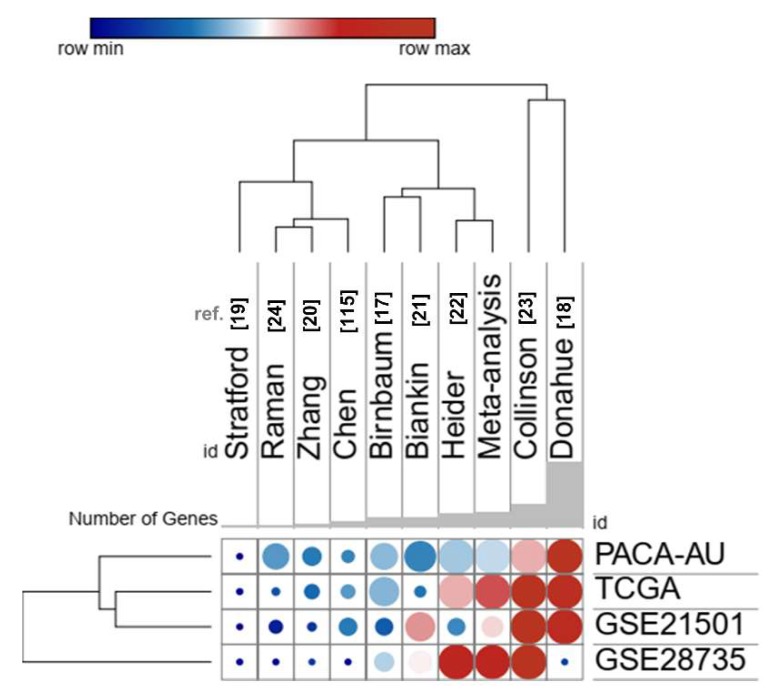
Performance of expression profile from the 39 secretome genes as potential pancreatic ductal adenocarcinoma (PDAC) biomarkers compared to nine previously gene signatures proposed for PDAC [17,18,19,20,21,22,23,24,110]. The color of the circles in the heat scatter plot represents the agreement index, while the size of the circle is based on the log-rank *p*-value of the risk group separation based on the SurvExpress tool. Rows and columns were grouped based on the Euclidean distance between the agreement index values. Datasets of PDAC patients: PDAC-TCGA [108], PACA-AU—ICGC [109], GSE21501 [19], and GSE28735 [20,79].

Several prognostic gene signatures for PDAC have been proposed [17,18,19,20,21,22,23,24,110]. However, we found no gene overlapping with our set of secreted-related genes with these previous signatures from the literature. We also compared the performance of our signature with nine gene expression signatures [17,18,19,20,21,22,23,24,110] in predicting worse survival in PDAC, which was tested in four different PDAC datasets (TCGA, ICGC, GSE21501, GSE28735) available in the SurvExpress tool. These signatures were able to separate risk-groups based on the gene expression profiles (data are not shown). The signatures proposed by Collisson et al. [23] and Donahue et al. [18] showed slightly better results than the set of secretome-related genes identified in our meta-analysis; however, Donahue’s signature, which corresponds to a set of 171 genes, had a lower performance in the dataset GSE28735 (Figure 8). Our signature also had comparable results with Haider et al. [22] (Figure 8). However, it is important to emphasize our dataset comprises proteins that are expressed and secreted in PDAC, constituting a rich source of biomarkers.

### 2.7. In Silico Validation of Secreted Proteins

**Figure 9:**
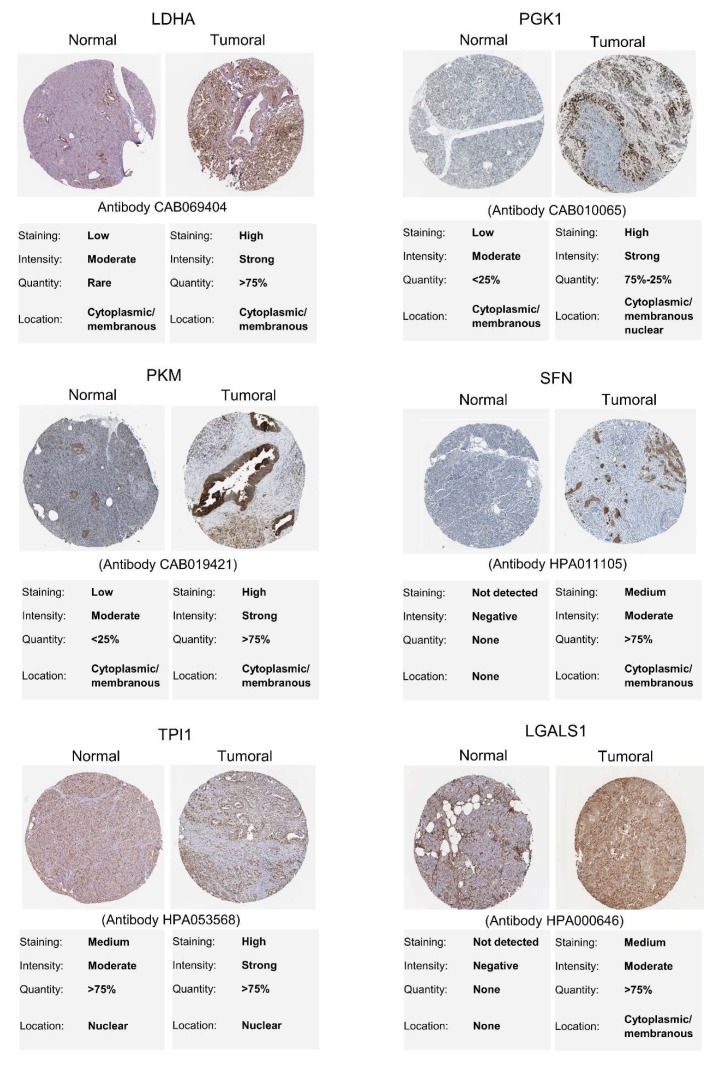
Identification of secreted proteins in normal and pancreatic ductal adenocarcinoma (PDAC) tissues using immunohistochemical staining data available at the Human Protein Atlas database [111]. These proteins were selected based on their significant increased gene expression, as identified using the GEPIA tool [102].

Immunohistochemical staining for 31 proteins identified with increased gene expression in PDAC tumoral samples by the GEPIA was retrieved from the HPA database [111]. PDAC immunohistochemical images were analyzed, and six proteins (L-lactate dehydrogenase A chain, LDHA; Phosphoglycerate kinase 1, PGK1; Pyruvate kinase, PKM; 14-3-3 protein sigma, SFN; Fibronectin, FN1; Galectin-1, LGALS1) showed medium or high immunostaining in PDAC tumor tissue, while low or not detected in normal pancreatic tissue, indicating that these proteins are also potentially biopsy-based markers to screening PDAC patients with high-risk (Figure 9 and Figure S4). Additionally, four proteins (Triosephosphate isomerase, TPI1; Galectin-3, LGALS3; Galectin-3-binding protein, LGALS3BP; Filamin-A, FLNA) showed average immunostaining in normal tissue and high in tumor tissue (Figure S4).

## 3. Discussion

Several studies have focused on the investigation of PDAC secretome-proteome in order to identify molecular mechanisms and biomarkers of this malignancy. However, the molecular complexity of pancreatic cancer and the failure of validation for most proposed biomarkers increase the urgency of effective strategies for identifying promising biomarkers of the disease. The heterogeneity of pancreatic cancer proteomic research using different samples, and fractionation and mass spectrometry techniques generates a rich source of datasets that can be explored, integrated, and compared to identify potential biomarkers of the disease. Here, we integrated the proteomic profiles of pancreatic cancer cell lines and tumors obtained by a meta-analysis of the secretome and proteome. This analysis identified 39 biologically relevant secreted proteins in PDAC. The gene expression profile of these proteins predicted worse overall survival in four independent cohorts of PDAC patients. The expression of these secretome-related genes also predicted worse overall survival in nine additional tumor types from the TCGA. Our meta-analysis approach using different proteomic datasets provided higher statistical power to address the biological heterogeneity of PDAC as well as overall patient survival. This strategy allowed us to identify a panel of proteins in pancreatic cancer proteomic studies, which are potential prognostic biomarkers.

Our approach also demonstrated the lack of overlap between the studies selected by the two meta-analysis strategies. We found no protein shared between all secretome and proteome studies. This discrepancy may be due to the diversity of proteomic techniques used by different studies; or limitations in proteomic analysis related to sample preparation and sample heterogeneity, proteome complexity to be analyzed, especially regarding protein expression levels; and limitations of the analytical methods [112,113,114,115]. To overcome these limitations, we selected the proteins presented in two or more studies, aiming to increase the possibility of detecting proteins involved in pancreatic cancer. We also integrated both secretome and proteome meta-analysis to select tumor proteins that are secreted or found in biological fluids. When we compared our list of 39 secretome proteins with PDAC gene signatures from the literature, we found no overlap between these previous studies [17,18,19,20,21,22,23,24,110]. This may be because global gene transcription levels insufficiently reflect global protein levels or due to limitations related to the sensibility of proteomics techniques, which point to the need to integrate transcriptomic and proteomic analyses to identify critical molecular changes of cancer in its essence [112,115,116,117,118]. Interestingly, our results show that tumor transcription levels of our set of proteins that are expressed and secreted in pancreatic cancer are useful as potential prognostic biomarkers when compared to previously proposed transcript-based signatures for PDAC.

We found a set of differentially expressed proteins with prognostic power that are biologically important in human cancers. For example, albumin (ALB), which was listed in 10 proteome and three secretome studies, has been identified as a poor prognostic factor in cancer patients [119,120]. Serum ALB is the most abundant blood protein in mammals; however, in diseases such as PDAC, its low level may be associated with an advanced stage of the disease [120]. The lower serum ALB levels can increase the risk of venous thromboembolism, which is the second leading cause of death in pancreatic cancer patients [119,121,122]. Triosephosphate Isomerase 1 (TPI1), found in seven secretome and four proteome studies, is a crucial enzyme in carbohydrate metabolism. Proteomic analysis of sera from pancreatic cancer patients showed TPI1 as one of the most abundant proteins in patients with poor survival before and after chemotherapy and could be further investigated as a prognostic marker as its levels gradually increase as the disease progresses [123]. The prognostic value TPI1 was also evaluated in gastric cancer, where patients with higher TPI1 expression had lower overall survival [124].

We also identified the alpha-enolase (ENO1), a glycolytic enzyme involved in the synthesis of pyruvate found in the cytoplasm, cell surface, and nucleus [125]. In addition to its glycolytic function, ENO1 plays a crucial role in cancer cell invasion and metastasis, in part because it also acts as a plasminogen receptor [126,127,128,129,130]. This ENO1 plasminogen receptor function, coupled with its high expression on the cell surface of tumor cells, facilitates the binding of large amounts of plasminogen on the cell surface, enabling plasmin activation, and enhancing the ability of PDAC cells to degrade extracellular matrix and, thus, benefiting the tumor invasion [127]. ENO1 also regulates pancreatic cancer adhesion, invasion, and metastasis by controlling the expression of αvβ3 integrin [128]. The αvβ3 integrin signaling is known to play a crucial role in tumor growth, angiogenesis, and metastasis. The protein αvβ3 integrin is the target of preclinical experiments in cancer treatment, demonstrating positive anti-angiogenic and anti-tumor effects [131,132]. These effects have been observed for cell surface ENO1, which has been found as significantly increased in tissues and plasma of PDAC patients with shorter survival [133]. Thus, further studies should be performed to investigate the role of secreted ENO1 in tumor biology. Additionally, ENO1 has also been seen as a potential prognostic biomarker in breast, head and neck cancers, and gliomas [125,134,135].

Our enrichment analysis also showed changes in the regulation of glycolytic processes. Increased aerobic glycolysis (Warburg effect) is observed exclusively in cancers and is highly dependent on unregulated metabolic enzymes [136]. In addition to the glycolytic enzymes ENO1 and TPI1, lactate dehydrogenase A (LDHA) was also identified in our study and corresponds to a central enzyme in regulating the Warburg effect; it catalyzes the conversion of lactate to pyruvate in the final stage of anaerobic glycolysis and is upregulated in various cancers [137,138]. In gastric cancer, LDHA was upregulated in tumor tissues and promoted tumor cell migration and invasion [137]. Moreover, LDHA has been reported to improve growth and inhibit apoptosis in pancreatic tumor cells [138]. Mohammad et al. [139] showed that increased expression of LDHA and PKM2 in pancreatic biopsy specimens from patients correlates with poor survival. Studies investigating the LDH expression levels in tumor and serum found that high tissue expression is not entirely consistent with elevated serum levels, indicating that tumor LDHA expression and serum levels are two independent predictors of the disease [140]. In our analysis, LDHA was identified in six secretome studies and only two from the proteome, suggesting that this enzyme may be secreted to perform its functions in tissues distant from the primary tumor focus, exacerbating the tumor progression. In addition to its prognostic role, further studies verifying the biological significance of serum LDHA levels need to be performed. Therefore, the high expression of glycolytic enzymes combined with their canonical and non-canonical functions may partly explain the aggressiveness of cancers, such as the pancreatic, influencing different prognostic outcomes and playing a pivotal role in defining personalized treatments [123,141].

Nucleoside diphosphate kinase A (NME1/NDPK-A) is a tumor suppressor with increased expression in different PDAC datasets and in other cancers types. The increased expression may be linked to anticancer mechanisms, as it exhibits anti-metastatic function [142]. Several studies support our findings, showing that NME1 is highly expressed in pancreatic cancer samples and predicts worse prognosis in patients [143,144,145]. Contrary to our results, Liu et al. [146], evaluated the prognostic value of NME1 by meta-analysis and concluded that negative regulation of NM1 was associated with a poor prognosis in breast, esophageal, nasopharyngeal, and lymphoma cancer. Negative regulation and worse prognosis of NME1 was also demonstrated in colon cancer [147]. Further investigations are needed to clarify the controversy between NME1 expression in other cancer types to address the relationship between expression and clinicopathological features.

Pancreatic cancer chemotherapeutic resistance is one of the characteristics that make it so aggressive. Among our protein pool, we found Galectin-1 (LGALS1), a glycan-binding protein that is highly expressed in PDAC stroma, and a significant contributor to pancreatic cancer progression [148]. Increased LGALS1 expression in cancer cells such as lung, liver, ovarian, glioblastoma, and lymphoma has been associated with drug resistance (temozolomide, sorafenib, rituximab, and cisplatin) [149,150,151,152,153,154,155,156]. Proteins with increased expression in cancers identified here, such as Galectin-3 (LGALS3), Peptidyl-prolyl cis-trans isomerase A (PPIA), Alpha-enolase (ENO1), L-lactate dehydrogenase A chain (LDHA), Protein/nucleic acid deglycase DJ-1 (PARK7), and Cathepsin D (CTSD), also confer resistance to chemotherapy [157,158,159,160,161,162,163,164,165]. These proteins use PI3K/AKT and AKT/NFKB pathways to inhibit apoptotic processes and promote survival in cancer cells [149,153,158,159,166,167]. Our results suggest that increased expression of secreted proteins identified in our integrative meta-analysis is also likely to be involved in the chemotherapy resistance processes, which in turn contributes to cancer progression and pancreatic cancer lethality.

Our meta-analysis used proteomic data, while our validation was performed with TCGA transcriptomic data. However, only one-third of the RNA species are significantly correlated with the corresponding proteins in human cells [168]. Thus, it is essential to emphasize that a combination of different factors influences a direct association between protein levels and their coding transcripts. These include the availability of a wide range of resources for protein biosynthesis, as well as temporal and spatial variations resulting from transcriptional and post-transcriptional mechanisms that control gene expression [26,169,170]. Previous integrated multi-platform analysis in PDAC has revealed associations of non-coding RNAs with tumor-specific mRNA subtypes [171]. These authors also showed that the differential regulation of gene expression via DNA methylation and microRNAs (miRNAs) could also distinguish tumor subtypes [171]. Recently, our research group has provided data on the role of miRNAs in the regulation of gene networks, including pathways of the adaptive and innate immune response involved in PDAC [172]. Thus, although transcript levels are not enough to predict protein levels in different conditions, the expression of 31 genes out of the 39 identified in our PDAC proteome-secretome meta-analysis was found upregulated in PDAC samples.

The transcripts and proteins identified in our study have the potential to be used in conjunction with miRNAs to increase the sensitivity and specificity of the PDAC diagnosis. To exemplify this point, the TIMP1 protein—which was identified in our study—has been indicated along with LCN2 as potential serum markers for the early detection of familial pancreatic cancer [173]. However, the combination of both LCN2 and TIMP1 with miR-196b was able to distinguish high-grade lesions and stage I from controls with absolute sensitivity and specificity [174]. Also, in accordance with our data, plasma extracellular vesicle long RNA profiling has identified a diagnostic signature, which includes the *TIMP1* transcript, for the detection of pancreatic cancer [175]. This increased detection of TIMP1 transcript and protein might be partially explained by the effects of miRNAs such as miR-221/222 and miR-21. These miRNAs are overexpressed in pancreatic cancer cells, and, as a result, they promote cellular proliferation, invasion, and chemoresistance by targeting TIMP-2 or inducing the expression of the invasion-related genes matrix metalloproteinase-2 and -9 [176,177].

The additional TCGA cancer types that we analyzed also shared many upregulated genes of the 39 secretome-related proteins, indicating that, at least in part, these tumors may share programs that promote oncogenesis and cancer progression. Some studies suggest that cancer may be categorized by gene and protein expression patterns of the secretome since similarities are observed between tumors originating from different tissues [134,178,179]. An explanation for these similarities is proposed by Robinson et al. [179], which shows a potential mechanism by which cancer cells relieve secretory stress by decreasing tissue-specific gene expression, facilitating the secretion of invasion-promoting proteins and proliferation. Recently, we have demonstrated that tumor types highly associated with cachexia share a high number of upregulated secretome genes [178]. These observations are in accordance with our findings since only *PRSS1* was explicitly expressed in the normal pancreas among the 39 proteins that we identified. However, it is important to note that *ARHGDIA* (Rho GDP-dissociation inhibitor 1) and *PARK7* (Protein/nucleic acid deglycase DJ-1) are specifically upregulated in PDAC, and the increased expression of *ARHGDIA* and *PARK7* can be considered as potential biomarkers of the disease. ARHGDIA—a specific regulator of Rho protein exchange reactions crucial for JNK pathway—was previously identified by a bioinformatic pipeline that searched for candidate genes related to pancreatic cancer using protein-protein interactions and a shortest path approach [180]. Also, in accordance with our results, PARK7 was found to be significantly elevated in PDAC [181], correlated with tumor invasion and worse patients’ outcome, and responsible for promoting invasion and metastasis of pancreatic cancer cells [182].

Our main contribution using this strategy consists of the selection of secreted proteins identified in pancreatic cancer proteomic analysis that stratify patients with low and high survival. However, our study has some limitations that should be pointed out, such as the reuse of proteomic data from studies with different biological samples, different protein fractionation approaches, and different mass spectrometry techniques employed. Another limitation of our investigation was the use of proteins present only in the body of the studies, not extending to supplementary data, which could broaden our range of identified proteins. We also noticed that some proteins identified in our analysis correspond to high abundance proteins in samples, suggesting that less abundant proteins may not have been identified by the proteomic techniques used in the studies retrieved by our meta-analysis. Also, our gene expression and survival analysis were performed in silico with the help of databases and bioinformatics tools available online, and experimental validation of the secreted proteins identified in this meta-analysis may help to correlate the results obtained herein with patient’s prognosis. Many of the observations made in this study were based on functions investigated at the cellular level, but the molecular mechanisms underlining the secretion of these proteins should be better studied, clarifying whether they are secreted to perform autocrine or paracrine functions, or if they have different functions and modes of action in different tissues. Thus, further research is needed to identify the molecular pathways and contributions of these secreted proteins in the pathophysiology of pancreatic cancer.

Importantly, the molecular subtyping of PDAC is in its infancy and remains without clinically relevant molecular subtypes [13]. Despite the extensive genomic characterization, gene signatures have provided limited prognostic information for the disease [183]. However, it is only by assessing the clinical importance of molecular subtypes that a relevant molecular profile may be identified [13]. 

## 4. Materials and Methods 

### 4.1. Integration of Secretome and Proteome Meta-Analysis to Identify Pancreatic Cancer Biomarkers

We integrated two meta-analyses to identify proteins as potential biomarkers of pancreatic cancer: a meta-analysis of pancreatic cancer secretome and meta-analysis of pancreatic ductal adenocarcinoma proteome. We searched protein data identified in these studies, published from 2005 to 2017, through the PubMed Central (PMC) electronic database at the U.S. National Institutes of Health’s National Library of Medicine (NIH/NLM). The study design of each meta-analysis followed the stages of the PRISMA statement [184].

#### 4.1.1. Pancreatic Cancer Secretome Meta-Analysis

To search for pancreatic cancer secretome studies, we used the following keywords: pancreatic cancer, pancreatic cancers, pancreatic neoplasm, pancreas cancer, pancreas cancers, cancer of the pancreas, cancer of pancreas and secretome, “cell secretome”, “cancer secretome”, “secretome analysis”, “secretome proteomic”, “secretome proteomics”, “secretome profiling”, “secretome mass spectrometry”, “microvesicles and proteome”, “microvesicles and proteomic”, “microvesicles and proteomics”, “microvesicles and protein profiling”, “microvesicles and mass spectrometry”, “exosome and proteome”, “exosome and proteomic”, “exosome and proteomics”, “exosome and protein profiling”, “exosome and mass spectrometry”, apoptotic bodies and proteome, apoptotic bodies and proteomic, apoptotic bodies and proteomics, apoptotic bodies and protein profiling, apoptotic bodies and spectrometry, extracellular vesicles and proteome, extracellular vesicles and proteomic, extracellular vesicles and proteomics, extracellular vesicles and proteomics, extracellular vesicles and protein profiling, extracellular vesicles and mass spectrometry, "secreted proteins" and proteome, "secreted proteins" and proteomic, "secreted proteins" and proteomics, "secreted proteins" and spectrometry mass, conditioned medium and proteome, conditioned medium and proteomic, conditioned medium and proteomics, "conditioned medium and protein profiling“, conditioned medium, and spectrometry mass. The criteria for study inclusion in this meta-analysis were: proteomic studies in pancreatic cancer samples (tumor tissue or pancreatic sulcus) or pancreatic cancer cell lines; only mass spectrometry studies were considered, and only data with statistical significance were included for the integrative analysis. Studies were excluded if they did not meet the criteria mentioned above, conducted in non-pancreatic cancer, reviews, studies with treatments prior to proteomic analysis, studies without proteomic analysis, in silico studies, unpublished studies, and studies published before 2005. As this analysis revealed that most studies involved in PDAC, we performed a meta-analysis of the proteome using only this histological type.

#### 4.1.2. Pancreatic Ductal Adenocarcinoma Proteome Meta-Analysis

To search for PDAC proteome studies, we used the following keywords: “pancreatic ductal adenocarcinoma” and proteome, “pancreatic ductal adenocarcinoma” and proteomic, “pancreatic ductal adenocarcinoma" and proteomics, “pancreatic ductal adenocarcinoma" and “protein profiling”, and “pancreatic ductal adenocarcinoma” and mass spectrometry. The following criteria were used to include studies in this meta-analysis: proteomic studies in human pancreatic ductal adenocarcinoma samples, only mass spectrometry studies were considered, studies that included normal tissues for comparison (case-control), and only data with statistical significance were included for the integrative analysis. Studies were excluded if they did not meet the criteria mentioned above or were studies on pancreatic ductal non-adenocarcinoma cancers, reviews, studies on experimental models, studies with treatments prior to proteomic analysis, studies without proteomic analysis, studies without case-control, studies without full access, in silico studies, protocols, and studies published before 2005.

### 4.2. Extraction of Meta-Analysis Data and in Silico Confirmation of Secreted Proteins

The studies were independently analyzed, and those that met the inclusion criteria were selected. The following information was extracted from each study: (1) study details: authors, year of publication, scientific journal, experimental model, mass spectrometry technique used, and validation strategies; (2) outcome measures: the proteins identified by the selected studies were tabulated to a Microsoft Excel file, mapped to the same gene symbol, and those identified simultaneously in two or more studies were selected for the study. The proteins shared between the two meta-analyses were identified by a Venn diagram tool (http://bioinfogp.cnb.csic.es/tools/venny/).

To confirm that all proteins commonly identified in the meta-analysis (secretome and proteome) are secreted, their amino acid sequences (FASTA file) were obtained from the UniProtKB database from the UniProt consortium. Next, the amino acid sequences or symbols of each protein were used to predict secreted proteins using the online tools SignalP 4.1, SecretomeP 2.0, TargetP 1.1, TMHMM v. 2.0, Vesiclepedia e Exocarta [94,95,96,97,99,100]. The SignalP 4.1 server has selected classically secreted proteins that have the signal peptide and D value above 0.45 [94]. The predicted proteins belonging to the non-classical secretion pathway without signal peptide were selected with the aid of the SecretomeP tool, and the cut-off point used was the neural network score (NN) >0.6 [95]. The Vesiclepedia and Exocarta databases were used to designate secreted proteins in exosomal fractions [96,97]. After secreted proteins were established by either the classical, nonclassical pathways or exosomes, and these proteins were challenged on the TargetP and TMHMM servers [99,100] for the exclusion of mitochondrial or transmembrane helix proteins, respectively.

### 4.3. Protein-Protein Interaction Network and Gene Ontology Analysis

The proteins identified as secreted were submitted to the STRING database (Search Tool for Retrieval of Interacting Genes, version 10.5; [101] for the construction of a protein-protein interaction network and analysis of ontology of pancreatic cancer secretome components. For the construction of networks, we consider experiments, databases, co-expression, neighborhood, and co-occurrence as sources of active interaction. The minimum interaction score required was 0.700 (high confidence), and nodes disconnected from the network were hidden to simplify the display. The PPI enrichment *p*-value indicates the statistical significance provided by STRING. For ontology analysis, we consider the top 15 terms with the lowest False Discovery Rate (FDR). Access in September 2018.

### 4.4. Gene Expression Profile in Pancreatic Ductal Adenocarcinoma

The transcriptional profile of genes encoding proteins identified in our meta-analysis as secreted in human pancreatic cancer was evaluated in 10 different cancers from the TCGA database [108] and compared with normal tissues from the TCGA and GTEx [104] databases after being uniformly processed and unified by Toil Pipeline [103] with the web-based Gene Expression Profiling Analysis tool (GEPIA) [102]. Differentially expressed genes between tumor samples and normal samples were determined by one-way ANOVA, applying the log2 fold-change > 1 and q-value <0.01 statistical cutoffs. These differentially expressed genes were further filtered for genes encoding predicted secreted proteins obtained from the HPA database (The Human Protein Atlas) [111], for 10 cancer types (PDAC, HNSCC, ESCC, GC, HCC, Lung AD, Lung SCC, COAD, AML, and BC) and compared with the set of secreted proteins identified by our meta-analysis. For data visualization, we constructed Heatmaps and performed Principal Component Analysis (PCA) in the ClustVis web tool [185]. Additionally, secreted proteins identified with corresponding increased gene expression in pancreatic tumor samples by GEPIA were submitted to the HPA database aiming at the immunohistochemical analysis of selected proteins in tumor and normal tissues.

### 4.5. Prognostic Value of Secreted Protein Translated Transcripts in the Predicting Pancreatic Ductal Adenocarcinoma Outcome

The SurvExpress database [107] was used for survival analysis and risk assessment in four different datasets of PDAC patients (PDAC-TCGA [108], PACA-AU—ICGC [109], GSE21501 [19], GSE28735 [20,79]), and in nine different cancers types from TCGA [108]. This tool allowed the association between the expression of the 39 secreted genes identified in pancreatic cancer with the survival of cancer patients using Cox Proportional Risk Regression, according to risk groups estimated by an optimization algorithm. Morpheus [186] was applied to select the best set of biomarkers from SurvExpress results, using a clustering analysis based on Euclidian distance.

## 5. Conclusions

Our integrative secretome and proteome meta-analysis in pancreatic cancer identified a set of 39 secreted proteins as potential biomarkers of the disease. The tumor gene expression profile of these 39 proteins predicted shorter survival in four different PDAC datasets (TCGA, ICGC, GSE21501, GSE28735) and nine different cancer types from the TCGA. The differential expression profile of this set of secreted proteins predicted worse overall survival in cancer patients and may also be used as potential therapeutic targets by acting on progression and resistance processes of pancreatic cancer.

## Figures and Tables

**Table 1:** Description of publicly available proteomic studies used in the secretome meta-analysis.

Ref.	Cell Line /Tumor Tissue	Labeling	Technique	Validation Method
[37]	Panc1	Silac	LC-MS/MS	WB, IHC, MA
[43]	AsPC1, MiaPaCa2, Panc1	Free	LC-MS/MS	-
[41]	Panc1	iTRAQ	LC-MS/MS	WB, ELISA
[45]	Paca44, Panc1, BxPc3, MiaPaca2, HPSC, A818-4	Free	LC-MS/MS	-
[46]	BxPC-3, MIA PaCa-2, Panc1, AsPC-1	Free	LC-MS/MS	WB, IHC, ELISA
[93]	CAPAN-2, RLT-PSC	Silac	LC-MRM/MS	-
[47]	PC-1.0, PC-1 (Hamster)	Silac	Nano-RPLC-MS/MS	WB
[49]	BxPC3, MiaPaca2, Panc1	Free	ESI-MS/MS	WB
[50]	SOJ-6, BxPC-3, MiaPaCa-2, Panc-1	Free	MALDI-TOF MS	WB
[34]	PAN02 (Mouse)	Free	MS/MS	ELISA
[51]	NIT-1	Free	MS/MS	WB
[52]	Panc-1	Free	LC-MS/MS	-
[53]	MiaPaCa-2, BxPc-3, Panc-1, AsPc-1	Free	MALDI–TOF MS	WB
[54]	Adenocarcinoma tissue	Free	LC-MS/MS	WB
[55]	BON-1, NCI-H727, SHP-77	Free	LC-MS/MS	WB
[3]	BxPc3, MIA-PaCa2, Panc1, CAPAN1, CFPAC1, SU.86.86, HPDE, PJ	Free	LC-MS/MS	ELISA
[56]	KLM, PK-59, MIAPaCa2	Free	MS/MS	WB
[57]	Panc-1, SW1990	iTRAQ	LC-MS/MS	-
[58]	MIA PaCa-2	labeling	MALDI-TOF MS	WB
[59]	CAPAN-2	Silac	LC-MS/MS	IHC

Ref.: reference; Rodent cell lines are specified in parentheses; Silac: Stable isotope labeling with amino acids in cell culture; iTRAQ: Isobaric tags for relative and absolute quantitation; LC-MS/MS: Liquid chromatography-tandem mass spectrometry; LC-MRM/MS: Multiplexed liquid chromatography multiple-reaction-monitoring mass spectrometry; Nano-RPLC-MS/MS: Nanoscale liquid chromatography coupled to tandem mass spectrometry; ESI-MS/MS: Electrospray ionization mass spectrometry; MALDI-TOF MS: Laser desorption ionization-time of flight mass spectrometry; MS/MS: Tandem mass spectrometry, PJ: Pancreatic juice; WB: Western Blot; IHC: Immunohistochemistry; MA: Microarrays.

**Table 2:** Description of publicly available human proteomic studies used in the proteome meta-analysis.

Ref.	Sample	Labeling	Technique	Validation Method
[6]	PTT	CD	nanoLC-ESI-MS/MS	IHC
[60]	PTT	CD	MALDI-TOF MS, nanoLC-ESI MS/MS	-
[61]	PTT	Free	MALDI-TOF/TOF MS, MS/MS	IHC
[62]	PTT	Free	MS/MS	WB
[63]	PTT	Free	MS/MS	WB, IHC
[64]	PTT	Free	LC-MS/MS	ELISA
[65]	PTT	Free	LC-MS/MS	ELISA
[66]	PTT	Free	MS/MS	-
[67]	PTT	Free	LC-MS/MS	IHC
[68]	PTT	Free	MALDI-TOF MS	ELISA
[69]	PTT	Free	LC-MS/MS	IHC
[70]	PTT	Free	MALDI-TOF MS	IHC, WB
[71]	PTT	CD	MALDI-TOF/TOF-MS	WB, IHC
[72]	PTT	Free	MALDI-TOF MS	WB
[73]	PTT	Free	MALDI-TOF/TOF-MS	-
[29]	PTT	Free	LC-MS/MS	IHC, WB
[74]	PTT	Free	LC-MS/MS	-
[75]	PTT	SI	LC-MS/MS	-
[76]	PTT	Free	LC-MS/MS	IHC, WB
[77]	PTT	LP	MS/MS	-
[78]	PTT	Free	LC-MS(MS)2	-
[79]	PTT	Free	UHPLC/MS/MS2	-
[80]	Urine	CD	MALDI-TOF MS	IB
[81]	Serum	Free	MALDI-QIT-TOF-MS	
[82]	Serum	Free	(MRM)	WB
[83]	Serum	Free	MS/MS	-
[84]	Blood	Free	MALDI-MS	ELISA
[85]	Serum	CD	MALDI-TOF/ TOF–MS	ELISA, WB
[86]	Serum	IL	LC-MS / MS	-
[87]	Urine	Free	LC/MS /MS	-
[88]	Plasma	Free	LC−MS/MS	ELISA, WB, IHC, RT-qPCR
[89]	PDF	Free	GeLC-MS/MS	-
[90]	Serum	Free	MALDI-TOF MS	PCR, WB, IHC
[91]	PJ	Free	MALDI-TOF MS MS/MS	WB, IHC, ELISA
[92]	Serum	Free	MALDI-TOF	WB

Ref.: Reference; PDF: Pancreatic duct fluid, PJ: Pancreatic juice; PTT: Pancreatic tumor tissue; SI: Stable Isotopic; IL: Isobaric Label; CD: CyDyes (Fluorescence Cyanine Dyes); LC-MS/MS: Liquid chromatography-tandem mass spectrometry; LC-MRM/MS: Multiplexed liquid chromatography multiple-reaction-monitoring mass spectrometry; Nano-RPLC-MS/MS: Nanoscale liquid chromatography coupled to tandem mass spectrometry; ESI-MS/MS: Electrospray ionization mass spectrometry; MALDI-TOF MS: Laser desorption ionization-time of flight mass spectrometry; MS/MS: Tandem mass spectrometry; MRM: Multiple Reaction Monitoring; IHC: Immunohistochemistry; WB: Western Blot; IB: Immunoblotting.

**Table 3:** Proteins shared between pancreatic cancer secretome and proteome studies.

Uniprot Access	Gene Symbol	Description
ALBU_HUMAN	ALB	Serum albumin
ENOA_HUMAN	ENO1	Alpha-enolase
FINC_HUMAN	FN1	Fibronectin
TRFE_HUMAN	TF	Serotransferrin
LEG1_HUMAN	LGALS1	Galectin-1
APOE_HUMAN	APOE	Apolipoprotein E
CATD_HUMAN	CTSD	Cathepsin D
TPIS_HUMAN	TPI1	Triosephosphate isomerase
GSTP1_HUMAN	GSTP1	Glutathione S-transferase P
PARK7_HUMAN	PARK7	Protein/nucleic acid deglycase DJ-1
TRY1_HUMAN	PRSS1	Trypsin-1
MOES_HUMAN	MSN	Moesin
PGK1_HUMAN	PGK1	Phosphoglycerate kinase 1
ANXA5_HUMAN	ANXA5	Annexin A5
PPIA_HUMAN	PPIA	Peptidyl-prolyl cis-trans isomerase A
KPYM_HUMAN	PKM	Pyruvate kinase PKM
EF1A1_HUMAN	EEF1A1	Elongation factor 1-alpha 1
TSP1_HUMAN	THBS1	Thrombospondin-1
GELS_HUMAN	GSN	Gelsolin
LEG3_HUMAN	LGALS3	Galectin-3
TIMP1_HUMAN	TIMP1	Metalloproteinase inhibitor 1
COF1_HUMAN	CFL1	Cofilin-1
FLNA_HUMAN	FLNA	Filamin-A
LG3BP_HUMAN	LGALS3BP	Galectin-3-binding protein
CALR_HUMAN	CALR	Calreticulin
CLIC1_HUMAN	CLIC1	Nuclear chloride ion channel protein
TAGL2_HUMAN	TAGLN2	Transgelin-2
LDHA_HUMAN	LDHA	L-lactate dehydrogenase A chain
NDKA_HUMAN	NME1	Nucleoside diphosphate kinase A
TKT_HUMAN	TKT	Transketolase
1433S_HUMAN	SFN	14-3-3 protein sigma
ALDOA_HUMAN	ALDOA	Fructose-bisphosphate aldolase A
ENOG_HUMAN	ENO2	Gamma-enolase
PGAM1_HUMAN	PGAM1	Phosphoglycerate mutase 1
GDIR1_HUMAN	ARHGDIA	Rho GDP-dissociation inhibitor 1
ACTB_HUMAN	ACTB	Actin, cytoplasmic 1
PDIA1_HUMAN	P4HB	Protein disulfide-isomerase
ACTS_HUMAN	ACTA1	Actin, alpha 1, skeletal muscle
FETUA_HUMAN	AHSG	Alpha-2-HS-glycoprotein

**Table 4:** Gene Ontology (GO) enrichment analysis of secreted proteins in pancreatic cancer.

**Cellular Component (GO)**			
**Pathway ID**	**Pathway Description**	**Gene Count**	**FDR**
GO.0070062	Extracellular exosome	35	1.29 × 10^24^
GO.0031988	Membrane-bounded vesicle	34	4.57 × 10^20^
GO.0005615	Extracellular space	25	1.79 × 10^19^
GO.0044421	Extracellular region part	31	3.35 × 10^15^
GO.0072562	Blood microparticle	11	1.15 × 10^14^
GO.0005576	Extracellular region	31	4.02 × 10^13^
GO.0060205	Cytoplasmic membrane-bounded vesicle lumen	8	4.29 × 10^10^
GO.0034774	Secretory granule lumen	6	2.35 × 10^7^
GO.0005925	Focal adhesion	9	1.88 × 10^6^
GO.0031093	Platelet alpha granule lumen	5	2.83 × 10^6^
GO.0031091	Platelet alpha granule	5	7.61 × 10^6^
GO.0044433	Cytoplasmic vesicle part	9	2.46 × 10^5^
GO.0005829	Cytosol	19	3.4 × 10^5^
GO.0030141	Secretory granule	7	7.44 × 10^5^
**Biological Process (GO)**			
**Pathway ID**	**Pathway Description**	**Gene Count**	**FDR**
GO.0006165	Nucleoside diphosphate phosphorylation	9	1.99 × 10^11^
GO.0006096	Glycolytic process	8	3.4 × 10^11^
GO.0002576	Platelet degranulation	9	5.25 × 10^11^
GO.0061621	Canonical glycolysis	7	5.25 × 10^11^
GO.0019674	NAD metabolic process	8	8.75 × 10^11^
GO.0046496	Nicotinamide nucleotide metabolic process	8	1.47 × 10^9^
GO.0030168	Platelet activation	10	5.73 × 10^9^
GO.0046364	Monosaccharide biosynthetic process	7	5.73 × 10^9^
GO.0009611	Response to wounding	14	9.81 × 10^9^
GO.0006090	Pyruvate metabolic process	7	1.09 × 10^8^
GO.0042981	Regulation of apoptotic process	17	1.51 × 10^8^
GO.0042060	Wound healing	13	3.84 × 10^8^
GO.0006094	Gluconeogenesis	6	8.46 × 10^8^
GO.0016192	Vesicle-mediated transport	15	1.23 × 10^7^
GO.0006950	Response to stress	23	1.39 × 10^7^
**Molecular Function (GO)**			
**Pathway ID**	**Pathway Description**	**Gene Count**	**FDR**
GO.0005515	Protein binding	24	0.000171
GO.0019899	Enzyme binding	12	0.00461
GO.0044822	Poly(A) RNA binding	11	0.0069
GO.0003723	RNA binding	12	0.0117
GO.0004634	Phosphopyruvate hydratase activity	2	0.0174

FDR: False discovery rate.

## Supplementary Materials

The following are available online at https://www.mdpi.com/2072-6694/12/3/716/s1, Figure S1: Expression profile of 39 secretome genes in normal and tumor tissues from GTEx (Genotype-Tissue Expression) and TCGA (The Cancer Genome Atlas), respectively, Figure S2: Principal component analysis (PCA) of 39 secretome genes in 10 different tumor types from TCGA compared to their respective normal tissues, Figure S3: The expression profile of 39 secretome genes by PDAC stratifies patients into low- and high-risk groups, Figure S4: Validation of secreted protein expression in normal and pancreatic ductal adenocarcinoma (PDAC) tissues using immunohistochemical staining available at the Human Protein Atlas database, Table S1: Total number of proteins identified in proteomic studies of pancreatic cancer, Table S2: Secretome proteins (*n* = 156) identified in two or more pancreatic cancer proteomic studies, Table S3: Proteins (*n* = 132) identified in two or more pancreatic cancer proteomic studies, Table S4: Selected studies and proteins included in the secretome and proteome pancreatic cancer meta-analyses, Table S5: Verification of secretion nature of 43 proteins, Table S6: The Cancer Genome Atlas (TCGA) and The Genotype-Tissue Expression (GTEx) samples used for gene expression analyses, Table S7: Expression of 39 secreted proteins based on samples from four different PDAC tumor data sets predicts poor overall survival, Table S8: Secretome genes with increased expression in high-risk groups from different datasets for PDAC in SurvExpress, Table S9: The expression of 39 secreted proteins based on The Cancer Genome Atlas (TCGA) tumor samples predicts poor overall survival.

## References

[1] Rahib L., Smith B.D., Aizenberg R., Rosenzweig A.B., Fleshman J.M., Matrisian L.M. (2014). Projecting Cancer Incidence and Deaths to 2030: The Unexpected Burden of Thyroid, Liver, and Pancreas Cancers in the United States. Cancer Res..

[2] Bray F., Ferlay J., Soerjomataram I., Siegel R.L., Torre L.A., Jemal A. (2018). Global cancer statistics 2018: GLOBOCAN estimates of incidence and mortality worldwide for 36 cancers in 185 countries. CA Cancer J. Clin..

[3] Makawita S., Smith C., Batruch I., Zheng Y., Rückert F., Grützmann R., Pilarsky C., Gallinger S., Diamandis E.P. (2011). Integrated Proteomic Profiling of Cell Line Conditioned Media and Pancreatic Juice for the Identification of Pancreatic Cancer Biomarkers. Mol. Cell. Proteom..

[4] Zhang M., Wang Z., Obazee O., Jia J., Childs E.J., Hoskins J., Figlioli G., Mocci E., Collins I., Chung C.C. (2016). Three new pancreatic cancer susceptibility signals identified on chromosomes 1q32.1, 5p15.33 and 8q24.21. Oncotarget.

[5] Jemal A., Siegel R., Ward E., Hao Y., Xu J., Thun M.J. (2009). Cancer Statistics, 2009. CA Cancer J. Clin..

[6] Sitek B., Sipos B., Alkatout I., Poschmann G., Stephan C., Schulenborg T., Marcus K., Lu J., Dittert D., Baretton G. (2009). Analysis of the Pancreatic Tumor Progression by a Quantitative Proteomic Approach and Immunhistochemical Validation research articles. J. Proteome Res..

[7] Kamisawa T., Wood L.D., Itoi T., Takaori K. (2016). Pancreatic cancer. Lancet.

[8] Yachida S., Jones S., Bozic I., Antal T., Leary R., Fu B., Kamiyama M., Hruban R.H., Eshleman J.R., Nowak M.A. (2010). Distant metastasis occurs late during the genetic evolution of pancreatic cancer. Nature.

[9] Liang C., Shi S., Meng Q., Liang D., Ji S., Zhang B., Qin Y., Xu J., Ni Q., Yu X. (2017). Complex roles of the stroma in the intrinsic resistance to gemcitabine in pancreatic cancer: Where we are and where we are going. Exp. Mol. Med..

[10] Chand S., O’Hayer K., Blanco F.F., Winter J.M., Brody J.R. (2016). The landscape of pancreatic cancer therapeutic resistance mechanisms. Int. J. Biol. Sci..

[11] Lewis R., Drebin J.A., Callery M.P., Fraker D., Kent T.S., Gates J., Vollmer C.M. (2013). A contemporary analysis of survival for resected pancreatic ductal adenocarcinoma. HPB.

[12] Ducreux M., Cuhna A.S., Caramella C., Hollebecque A., Burtin P., Goéré D., Seufferlein T., Haustermans K., Van Laethem J.L., Conroy T. (2015). Cancer of the pancreas: ESMO Clinical Practice Guidelines for diagnosis, treatment and follow-up. Ann. Oncol..

[13] Collisson E.A., Bailey P., Chang D.K., Biankin A.V. (2019). Molecular subtypes of pancreatic cancer. Nat. Rev. Gastroenterol. Hepatol..

[14] Kern S.E., Shi C., Hruban R.H. (2011). The complexity of pancreatic ductal cancers and multidimensional strategies for therapeutic targeting. J. Pathol..

[15] Kleeff J., Korc M., Apte M., La Vecchia C., Johnson C.D., Biankin A.V., Neale R.E., Tempero M., Tuveson D.A., Hruban R.H. (2016). Pancreatic cancer. Nat. Rev. Dis. Prim..

[16] Fong Z.V., Winter J.M. (2012). Biomarkers in Pancreatic Cancer. Cancer J..

[17] Birnbaum D.J., Finetti P., Lopresti A., Gilabert M., Poizat F., Raoul J.L., Delpero J.R., Moutardier V., Birnbaum D., Mamessier E. (2017). A 25-gene classifier predicts overall survival in resectable pancreatic cancer. BMC Med..

[18] Donahue T.R., Tran L.M., Hill R., Li Y., Kovochich A., Calvopina J.H., Patel S.G., Wu N., Hindoyan A., Farrell J.J. (2012). Integrative Survival-Based Molecular Profiling of Human Pancreatic Cancer. Clin. Cancer Res..

[19] Stratford J.K., Bentrem D.J., Anderson J.M., Fan C., Volmar K.A., Marron J.S., Routh E.D., Caskey L.S., Samuel J.C., Der C.J. (2010). A Six-Gene Signature Predicts Survival of Patients with Localized Pancreatic Ductal Adenocarcinoma. PLoS Med..

[20] Zhang G., Schetter A., He P., Funamizu N., Gaedcke J., Ghadimi B.M., Ried T., Hassan R., Yfantis H.G., Lee D.H. (2012). DPEP1 Inhibits Tumor Cell Invasiveness, Enhances Chemosensitivity and Predicts Clinical Outcome in Pancreatic Ductal Adenocarcinoma. PLoS ONE.

[21] Biankin A.V., Waddell N., Kassahn K.S., Gingras M.-C., Muthuswamy L.B., Johns A.L., Miller D.K., Wilson P.J., Patch A.-M., Wu J. (2012). Pancreatic cancer genomes reveal aberrations in axon guidance pathway genes. Nature.

[22] Haider S., Wang J., Nagano A., Desai A., Arumugam P., Dumartin L., Fitzgibbon J., Hagemann T., Marshall J.F., Kocher H.M. (2014). A multi-gene signature predicts outcome in patients with pancreatic ductal adenocarcinoma. Genome Med..

[23] Collisson E.A., Sadanandam A., Olson P., Gibb W.J., Truitt M., Gu S., Cooc J., Weinkle J., Kim G.E., Jakkula L. (2011). Subtypes of pancreatic ductal adenocarcinoma and their differing responses to therapy. Nat. Med..

[24] Raman P., Maddipati R., Lim K.H., Tozeren A. (2018). Pancreatic cancer survival analysis defines a signature that predicts outcome. PLoS ONE.

[25] Pradet-Balade B., Boulmé F., Beug H., Müllner E.W., Garcia-Sanz J.A. (2001). Translation control: Bridging the gap between genomics and proteomics?. Trends Biochem. Sci..

[26] Liu Y., Beyer A., Aebersold R. (2016). On the Dependency of Cellular Protein Levels on mRNA Abundance. Cell.

[27] Hanash S., Taguchi A. (2010). The grand challenge to decipher the cancer proteome. Nat. Rev. Cancer.

[28] Domon B., Aebersold R. (2006). Challenges and Opportunities in Proteomics Data Analysis. Mol. Cell. Proteom..

[29] Iuga C., Seicean A., Iancu C., Buiga R., Sappa P.K., Völker U., Hammer E. (2014). Proteomic identification of potential prognostic biomarkers in resectable pancreatic ductal adenocarcinoma. Proteomics.

[30] Jimenez-Luna C., Torres C., Ortiz R., Dieguez C., Martinez-Galan J., Melguizo C., Prados J.C., Caba O. (2018). Proteomic biomarkers in body fluids associated with pancreatic cancer. Oncotarget.

[31] Brandi J., Manfredi M., Speziali G., Gosetti F., Marengo E., Cecconi D. (2018). Proteomic approaches to decipher cancer cell secretome. Semin. Cell Dev. Biol..

[32] Donadelli M. (2018). The cancer secretome and secreted biomarkers. Semin. Cell Dev. Biol..

[33] Houg D.S., Bijlsma M.F. (2018). The hepatic pre-metastatic niche in pancreatic ductal adenocarcinoma. Mol. Cancer.

[34] Costa-Silva B., Aiello N.M., Ocean A.J., Singh S., Zhang H., Thakur B.K., Becker A., Hoshino A., Mark M.T., Molina H. (2015). Pancreatic cancer exosomes initiate pre-metastatic niche formation in the liver. Nat. Cell Biol..

[35] Melo S., Luecke L.B., Kahlert C., Fernandez A.F., Gammon S.T., Kaye J., Lebleu V.S., Mittendorf E.A., Weitz J., Rahbari N. (2015). Glypican-1 identifies cancer exosomes and detects early pancreatic cancer. Nature.

[36] Lobb R.J., Lima L.G., Möller A. (2017). Exosomes: Key mediators of metastasis and pre-metastatic niche formation. Semin. Cell Dev. Biol..

[37] Grønborg M., Kristiansen T.Z., Iwahori A., Chang R., Reddy R., Sato N., Molina H., Jensen O.N., Hruban R.H., Goggins M.G. (2006). Biomarker Discovery from Pancreatic Cancer Secretome Using a Differential Proteomic Approach. Mol. Cell. Proteom..

[38] Belczacka I., Latosinska A., Metzger J., Marx D., Vlahou A., Mischak H., Frantzi M. (2018). Proteomics biomarkers for solid tumors: Current status and future prospects. Mass Spectrom. Rev..

[39] Makridakis M., Vlahou A. (2010). Secretome proteomics for discovery of cancer biomarkers. J. Proteom..

[40] Pavlou M.P., Diamandis E.P. (2010). The cancer cell secretome: A good source for discovering biomarkers?. J. Proteom..

[41] Brandi J., Pozza E.D., Dando I., Biondani G., Robotti E., Jenkins R., Elliott V., Park K., Marengo E., Costello E. (2016). Secretome protein signature of human pancreatic cancer stem-like cells. J. Proteom..

[42] Ray P., Rialon-Guevara K.L., Veras E., Sullenger B.A., White R.R. (2012). Comparing human pancreatic cell secretomes by in vitro aptamer selection identifies cyclophilin B as a candidate pancreatic cancer biomarker. J. Clin. Investig..

[43] Schiarea S., Solinas G., Allavena P., Scigliuolo G.M., Bagnati R., Fanelli R., Chiabrando C. (2010). Secretome analysis of multiple pancreatic cancer cell lines reveals perturbations of key functional networks. J. Proteome Res..

[44] Xue H., Lu B., Lai M. (2008). The cancer secretome: A reservoir of biomarkers. J. Transl. Med..

[45] Klein-Scory S., Tehrani M.M., Eilert-Micus C., Adamczyk K.A., Wojtalewicz N., Schnölzer M., Hahn S.A., Schmiegel W., Schwarte-Waldhoff I. (2014). New insights in the composition of extracellular vesicles from pancreatic cancer cells: Implications for biomarkers and functions. Proteome Sci..

[46] Chang Y.T., Wu C.C., Shyr Y.M., Chen T.C., Hwang T.L., Yeh T.S., Chang K.P., Liu H.P., Liu Y.L., Tsai M.H. (2011). Secretome-based identification of ULBP2 as a novel serum marker for pancreatic cancer detection. PLoS ONE.

[47] Liu P., Weng Y., Sui Z., Wu Y., Meng X., Wu M., Jin H., Tan X., Zhang L., Zhang Y. (2016). Quantitative secretomic analysis of pancreatic cancer cells in serum-containing conditioned medium. Sci. Rep..

[48] Borrebaeck C.A.K. (2017). Precision diagnostics: Moving towards protein biomarker signatures of clinical utility in cancer. Nat. Rev. Cancer.

[49] Adamczyk K.A., Klein-Scory S., Tehrani M.M., Warnken U., Schmiegel W., Schnölzer M., Schwarte-Waldhoff I. (2011). Characterization of soluble and exosomal forms of the EGFR released from pancreatic cancer cells. Life Sci..

[50] Ristorcelli E., Beraud E., Verrando P., Villard C., Lafitte D., Sbarra V., Lombardo D., Verine A. (2008). Human tumor nanoparticles induce apoptosis of pancreatic cancer cells. FASEB J..

[51] Hyo S.L., Jeong J., Lee K.J. (2009). Characterization of vesicles secreted from insulinoma NIT-1 cells. J. Proteome Res..

[52] Que R., Lin C., Ding G.-P., Wu Z.-R., Cao L.-P. (2016). Increasing the immune activity of exosomes: The effect of miRNA-depleted exosome proteins on activating dendritic cell/cytokine-induced killer cells against pancreatic cancer. J. Zhejiang Univ. B.

[53] Walsh N., Dowling P., O’Donovan N., Henry M., Meleady P., Clynes M. (2008). Aldehyde dehydrogenase 1A1 and gelsolin identified as novel invasion-modulating factors in conditioned medium of pancreatic cancer cells. J. Proteom..

[54] McKinney K.Q., Lee Y.Y., Choi H.S., Groseclose G., Iannitti D.A., Martinie J.B., Russo M.W., Lundgren D.H., Han D.K., Bonkovsky H.L. (2011). Discovery of putative pancreatic cancer biomarkers using subcellular proteomics. J. Proteom..

[55] Srirajaskanthan R., Caplin M.E., Waugh M.G., Watkins J., Meyer T., Hsuan J.J., Beaumont N.J. (2010). Identification of Mac-2-binding protein as a putative marker of neuroendocrine tumors from the analysis of cell line secretomes. Mol. Cell. Proteom..

[56] Baron B., Kitagawa T., Nakamura K., Kuramitsu Y. (2015). Isolation of a growth factor stress-induced pancreatic cancer sub-population: Insight into changes due to micro-environment. Cancer Genom. Proteom..

[57] Zhang H., Lv L., Liu H., Cui L., Chen G., Bi P., Li Z. (2009). Profiling the potential biomarkers for cell differentiation of pancreatic cancer using iTRAQ and 2-D LC-MS/MS. Proteom. Clin. Appl..

[58] Xiao J., Lee W., Zhao Y., Cao R., Go V. (2010). Profiling Pancreatic Cancer–Secreted Proteome Using 15N Amino Acids and Serum-Free Media. Pancreas.

[59] Yu K.H., Barry C.G., Austin D., Busch C.M., Sangar V., Rustgi A.K., Blair I.A. (2009). Stable isotope dilution multidimensional liquid chromatography-tandem mass spectrometry for pancreatic cancer serum biomarker discovery. J. Proteome Res..

[60] Sitek B., Luttges J., Marcus K., Kloppel G., Schmiegel W., Meyer H.E., Hahn S.A., Stuhler K. (2005). Application of fluorescence difference gel electrophoresis saturation labelling for the analysis of microdissected precursor lesions of pancreatic ductal adenocarcinoma. Proteomics.

[61] Qi T., Han J., Cui Y., Zong M., Liu X., Zhu B. (2008). Comparative proteomic analysis for the detection of biomarkers in pancreatic ductal adenocarcinomas. J. Clin. Pathol..

[62] Turtoi A., Musmeci D., Wang Y.H., Dumont B., Somja J., Bevilacqua G., De Pauw E., Delvenne P., Castronovo V. (2011). Identification of Novel Accessible Proteins Bearing Diagnostic and Therapeutic Potential in Human Pancreatic Ductal Adenocarcinoma. J. Proteome Res..

[63] Satoh M., Takano S., Sogawa K., Noda K., Yoshitomi H., Ishibashi M., Mogushi K., Takizawa H., Otsuka M., Shimizu H. (2017). Immune-complex level of cofilin-1 in sera is associated with cancer progression and poor prognosis in pancreatic cancer. Cancer Sci..

[64] Lin C., Wu W.-C., Zhao G.-C., Wang D.-S., Lou W.-H., Jin D.-Y. (2016). ITRAQ-based quantitative proteomics reveals apolipoprotein A-I and transferrin as potential serum markers in CA19-9 negative pancreatic ductal adenocarcinoma. Medicine.

[65] Kosanam H., Prassas I., Chrystoja C.C., Soleas I., Chan A., Dimitromanolakis A., Blasutig I.M., Rückert F., Gruetzmann R., Pilarsky C. (2013). Laminin, gamma 2 (LAMC2): A Promising New Putative Pancreatic Cancer Biomarker Identified by Proteomic Analysis of Pancreatic Adenocarcinoma Tissues. Mol. Cell. Proteom..

[66] Mayerle J., Kalthoff H., Reszka R., Kamlage B., Peter E., Schniewind B., Maldonado S.G., Pilarsky C., Heidecke C.D., Schatz P. (2017). Metabolic biomarker signature to differentiate pancreatic ductal adenocarcinoma from chronic pancreatitis. Gut.

[67] Takadate T., Onogawa T., Fukuda T., Motoi F., Suzuki T., Fujii K., Kihara M., Mikami S., Bando Y., Maeda S. (2013). Novel prognostic protein markers of resectable pancreatic cancer identified by coupled shotgun and targeted proteomics using formalin-fixed paraffin-embedded tissues. Int. J. Cancer.

[68] Hwang T.-L., Liang Y., Chien K.-Y., Yu J.-S. (2006). Overexpression and elevated serum levels of phosphoglycerate kinase 1 in pancreatic ductal adenocarcinoma. Proteomics.

[69] Kuwae Y., Kakehashi A., Wakasa K., Wei M. (2014). Paraneoplastic Ma Antigen—Like 1 as a Potential Prognostic. Pancreas.

[70] Tian R., Wei L.-M., Qin R.-Y., Li Y., Du Z.-Y., Xia W., Shi C.-J., Jin H. (2008). Proteome analysis of human pancreatic ductal adenocarcinoma tissue using two-dimensional gel electrophoresis and tandem mass spectrometry for identification of disease-related proteins. Dig. Dis. Sci..

[71] Cui Y., Tian M., Zong M., Teng M., Chen Y., Lu J., Jiang J., Liu X., Han J. (2009). Proteomic analysis of pancreatic ductal adenocarcinoma compared with normal adjacent pancreatic tissue and pancreatic benign cystadenoma. Pancreatology.

[72] Chung J.C., Oh M.J., Choi S.H., Bae C.D. (2008). Proteomic analysis to identify biomarker proteins in pancreatic ductal adenocarcinoma. ANZ J. Surg..

[73] Cui Y., Zhang D., Jia Q., Li T., Zhang W., Han J. (2009). Proteomic and tissue array profiling identifies elevated hypoxia-regulated proteins in pancreatic ductal adenocarcinoma. Cancer Investig..

[74] Britton D., Zen Y., Quaglia A., Selzer S., Mitra V., Löbner C., Jung S., Böhm G., Schmid P., Prefot P. (2014). Quantification of pancreatic cancer proteome and phosphorylome: Indicates molecular events likely contributing to cancer and activity of drug targets. PLoS ONE.

[75] Pan S., Chen R., Tamura Y., Crispin D.A., Lai L.A., May D.H., McIntosh M.W., Goodlett D.R., Brentnall T. (2014). A Quantitative Glycoproteomics Analysis Reveals Changes in N-Glycosylation Level Associated with Pancreatic Ductal Adenocarcinoma. J. Proteome Res..

[76] Kawahara T., Hotta N., Ozawa Y., Kato S., Kano K., Yokoyama Y., Nagino M., Takahashi T., Yanagisawa K. (2013). Quantitative proteomic profiling identifies DPYSL3 as pancreatic ductal adenocarcinoma-associated molecule that regulates cell adhesion and migration by stabilization of focal adhesion complex. PLoS ONE.

[77] Chen R., Pan S., Ottenhof N.A., de Wilde R.F., Wolfgang C.L., Lane Z., Post J., Bronner M.P., Willmann J.K., Maitra A. (2012). Stromal galectin-1 expression is associated with long-term survival in resectable pancreatic ductal adenocarcinoma. Cancer Biol. Ther..

[78] Kojima K., Bowersock G.J., Kojima C., Klug C.A., Grizzle W.E., Mobley J.A. (2012). Validation of a robust proteomic analysis carried out on formalin-fixed paraffin-embedded tissues of the pancreas obtained from mouse and human. Proteomics.

[79] Zhang G., He P., Tan H., Budhu A., Gaedcke J., Michael Ghadimi B., Ried T., Yfantis H.G., Lee D.H., Maitra A. (2013). Integration of metabolomics and transcriptomics revealed a fatty acid network exerting growth inhibitory effects in human pancreatic cancer. Clin. Cancer Res..

[80] Weeks M.E., Hariharan D., Petronijevic L., Radon T.P., Whiteman H.J., Kocher H.M., Timms J.F., Lemoine N.R., Crnogorac-Jurcevic T. (2008). Analysis of the urine proteome in patients with pancreatic ductal adenocarcinoma. Proteom. Clin. Appl..

[81] Chen J., Anderson M., Misek D.E., Simeone D.M., Lubman D.M. (2007). Characterization of apolipoprotein and apolipoprotein precursors in pancreatic cancer serum samples via two-dimensional liquid chromatography and mass spectrometry. J. Chromatogr. A.

[82] Jenkinson C., Elliott V.L., Evans A., Oldfield L., Jenkins R.E., O’brien D.P., Apostolidou S., Gentry-Maharaj A., Fourkala E.O., Jacobs I.J. (2016). Decreased serum thrombospondin-1 levels in pancreatic cancer patients up to 24 months prior to clinical diagnosis: Association with diabetes mellitus. Clin. Cancer Res..

[83] Hocker J.R., Postier R.G., Li M., Lerner M.R., Lightfoot S.A., Peyton M.D., Deb S.J., Baker C.M., Williams T.L., Hanas R.J. (2015). Discriminating patients with early-stage pancreatic cancer or chronic pancreatitis using serum electrospray mass profiling. Cancer Lett..

[84] Guo X., Lv X., Fang C., Lv X., Wang F., Wang D., Zhao J., Ma Y., Xue Y., Bai Q. (2016). Dysbindin as a novel biomarker for pancreatic ductal adenocarcinoma identified by proteomic profiling. Int. J. Cancer.

[85] Chen J., Chen L.J., Xia Y.L., Zhou H.C., Yang R.B., Wu W., Lu Y., Hu L.W., Zhao Y. (2013). Identification and verification of transthyretin as a potential biomarker for pancreatic ductal adenocarcinoma. J. Cancer Res. Clin. Oncol..

[86] Sogawa K., Takano S., Iida F., Satoh M., Tsuchida S., Kawashima Y., Yoshitomi H., Sanda A., Kodera Y., Takizawa H. (2016). Identification of a novel serum biomarker for pancreatic cancer, C4b-binding protein α-chain (C4BPA) by quantitative proteomic analysis using tandem mass tags. Br. J. Cancer.

[87] Radon T.P., Massat N.J., Jones R., Alrawashdeh W., Dumartin L., Ennis D., Duffy S.W., Kocher H.M., Pereira S.P., Guarner L. (2015). Identification of a three-biomarker panel in urine for early detection of pancreatic adenocarcinoma. Clin. Cancer Res..

[88] Lee M.J., Na K., Jeong S.-K., Lim J.-S., Kim S.A., Lee M.-J., Song S.Y., Kim H., Hancock W.S., Paik Y.-K. (2014). Identification of Human Complement Factor B as a Novel Biomarker Candidate for Pancreatic Ductal Adenocarcinoma. J. Proteome Res..

[89] Chen K.T., Kim P.D., Jones K.A., Devarajan K., Patel B.B., Hoffman J.P., Ehya H., Huang M., Watson J.C., Tokar J.L. (2014). Potential Prognostic Biomarkers of Pancreatic Cancer. Pancreas.

[90] Chen J., Wu W., Chen L., Zhou H., Yang R., Hu L., Zhao Y. (2013). Profiling the potential tumor markers of pancreatic ductal adenocarcinoma using 2D-DIGE and MALDI-TOF-MS: Up-regulation of Complement C3 and alpha-2-HS-glycoprotein. Pancreatology.

[91] Tian M., Cui Y.-Z., Song G.-H., Zong M.-J., Zhou X.-Y., Chen Y., Han J.-X. (2008). Proteomic analysis identifies MMP-9, DJ-1 and A1BG as overexpressed proteins in pancreatic juice from pancreatic ductal adenocarcinoma patients. BMC Cancer.

[92] Tomaino B., Cappello P., Capello M., Fredolini C., Ponzetto A., Novarino A., Ciuffreda L., Bertetto O., De Angelis C., Gaia E. (2007). Autoantibody signiture in human ductal pancreatic adenocarcinoma. J. Proteome Res..

[93] Wehr A.Y., Hwang W.T., Blair I.A., Yu K.H. (2012). Relative quantification of serum proteins from pancreatic ductal adenocarcinoma patients by stable isotope dilution liquid chromatography-mass spectrometry. J. Proteome Res..

[94] Petersen T.N., Brunak S., von Heijne G., Nielsen H. (2011). SignalP 4.0: Discriminating signal peptides from transmembrane regions. Nat. Methods.

[95] Bendtsen J.D., Jensen L.J., Blom N., Von Heijne G., Brunak S. (2004). Feature-based prediction of non-classical and leaderless protein secretion. Protein Eng. Des. Sel..

[96] Kalra H., Simpson R.J., Ji H., Aikawa E., Altevogt P., Askenase P., Bond V.C., Borràs F.E., Breakefield X., Budnik V. (2012). Vesiclepedia: A Compendium for Extracellular Vesicles with Continuous Community Annotation. PLoS Biol..

[97] Mathivanan S., Fahner C.J., Reid G.E., Simpson R.J. (2012). ExoCarta 2012: Database of exosomal proteins, RNA and lipids. Nucleic Acids Res..

[98] Nanjappa V., Thomas J.K., Marimuthu A., Muthusamy B., Radhakrishnan A., Sharma R., Ahmad Khan A., Balakrishnan L., Sahasrabuddhe N.A., Kumar S. (2014). Plasma Proteome Database as a resource for proteomics research: 2014 update. Nucleic Acids Res..

[99] Emanuelsson O., Nielsen H., Brunak S., von Heijne G. (2000). Predicting Subcellular Localization of Proteins Based on their N-terminal Amino Acid Sequence. J. Mol. Biol..

[100] Krogh A., Larsson B., von Heijne G., Sonnhammer E.L. (2001). Predicting transmembrane protein topology with a hidden markov model: Application to complete genomes. J. Mol. Biol..

[101] Szklarczyk D., Morris J.H., Cook H., Kuhn M., Wyder S., Simonovic M., Santos A., Doncheva N.T., Roth A., Bork P. (2017). The STRING database in 2017: Quality-controlled protein-protein association networks, made broadly accessible. Nucleic Acids Res..

[102] Tang Z., Li C., Kang B., Gao G., Li C., Zhang Z. (2017). GEPIA: A web server for cancer and normal gene expression profiling and interactive analyses. Nucleic Acids Res..

[103] Vivian J., Rao A.A., Nothaft F.A., Ketchum C., Armstrong J., Novak A., Pfeil J., Narkizian J., Deran A.D., Musselman-Brown A. (2017). Toil enables reproducible, open source, big biomedical data analyses. Nat. Biotechnol..

[104] Lonsdale J., Thomas J., Salvatore M., Phillips R., Lo E., Shad S., Hasz R., Walters G., Garcia F., Young N. (2013). The Genotype-Tissue Expression (GTEx) project. Nat. Genet..

[105] Lowenfels A.B., Maisonneuve P., DiMagno E.P., Elitsur Y., Gates L.K., Perrault J., Whitcomb D.C. (1997). Hereditary pancreatitis and the risk of pancreatic cancer. International Hereditary Pancreatitis Study Group. J. Natl. Cancer Inst..

[106] Lowenfels A.B., Maisonneuve P., Cavallini G., Ammann R.W., Lankisch P.G., Andersen J.R., Dimagno E.P., Andren-Sandberg A., Domellof L. (1993). Pancreatitis and the Risk of Pancreatic Cancer. N. Engl. J. Med..

[107] Aguirre-Gamboa R., Gomez-Rueda H., Martínez-Ledesma E., Martínez-Torteya A., Chacolla-Huaringa R., Rodriguez-Barrientos A., Tamez-Peña J.G., Treviño V. (2013). SurvExpress: An online biomarker validation tool and database for cancer gene expression data using survival analysis. PLoS ONE.

[108] Tomczak K., Czerwińska P., Wiznerowicz M. (2014). The Cancer Genome Atlas (TCGA ): An immeasurable source of knowledge. Contemp. Oncol..

[109] International Cancer Genome Consortium T.I.C.G., Hudson T.J., Anderson W., Artez A., Barker A.D., Bell C., Bernabé R.R., Bhan M.K., Calvo F., Eerola I. (2010). International network of cancer genome projects. Nature.

[110] Chen D.T., Davis-Yadley A.H., Huang P.Y., Husain K., Centeno B.A., Permuth-Wey J., Pimiento J.M., Malafa M. (2015). Prognostic fifteen-gene signature for early stage pancreatic ductal adenocarcinoma. PLoS ONE.

[111] Uhlen M., Oksvold P., Fagerberg L., Lundberg E., Jonasson K., Forsberg M., Zwahlen M., Kampf C., Wester K., Hober S. (2010). Towards a knowledge-based Human Protein Atlas. Nat. Biotechnol..

[112] Garbis S., Lubec G., Fountoulakis M. (2005). Limitations of current proteomics technologies. J. Chromatogr. A.

[113] Reymond M.A., Schlegel W. (2007). Proteomics in cancer. Adv. Clin. Chem..

[114] Lim L.C., Lim Y.M. (2018). Proteome Heterogeneity in Colorectal Cancer. Proteomics.

[115] Bateman N.W., Conrads T.P. (2018). Recent Advances and Opportunities in Proteomic Analyses of Tumor Heterogeneity. J. Pathol..

[116] Anderson L., Seilhamer J. (1997). A comparison of selected mRNA and protein abundances in human liver. Electrophoresis.

[117] Gygi S.P., Rochon Y., Franza B.R., Aebersold R. (1999). Correlation between Protein and mRNA Abundance in Yeast. Mol. Cell. Biol..

[118] Hsiao Y.C., Chu L.J., Chen J.T., Yeh T.S., Yu J.S. (2017). Proteomic profiling of the cancer cell secretome: Informing clinical research. Expert Rev. Proteom..

[119] Konigsbrugge O., Posch F., Riedl J., Reitter E.-M., Zielinski C., Pabinger I., Ay C. (2016). Association Between Decreased Serum Albumin With Risk of Venous Thromboembolism and Mortality in Cancer Patients. Oncologist.

[120] Deng Q.L., Dong S., Wang L., Zhang C.Y., Ying H.F., Li Z.S., Shen X.H., Guo Y.B., Meng Z.Q., Yu J.M. (2017). Development and Validation of a Nomogram for Predicting Survival in Patients with Advanced Pancreatic Ductal Adenocarcinoma. Sci. Rep..

[121] Menapace L.A., Peterson D.R., Berry A., Sousou T., Khorana A.A. (2011). Symptomatic and incidental thromboembolism are both associated with mortality in pancreatic cancer. Thromb. Haemost..

[122] Kondo S., Sasaki M., Hosoi H., Sakamoto Y., Morizane C., Ueno H., Okusaka T. (2018). Incidence and risk factors for venous thromboembolism in patients with pretreated advanced pancreatic carcinoma. Oncotarget.

[123] Follia L., Ferrero G., Mandili G., Beccuti M., Giordano D., Spadi R., Satolli M.A., Evangelista A., Katayama H., Hong W. (2019). Integrative Analysis of Novel Metabolic Subtypes in Pancreatic Cancer Fosters New Prognostic Biomarkers. Front. Oncol..

[124] Chen T., Huang Z., Tian Y., Lin B., He R., Wang H., Ouyang P., Chen H., Wu L. (2017). Clinical significance and prognostic value of Triosephosphate isomerase expression in gastric cancer. Medicine.

[125] Song Y., Luo Q., Long H., Hu Z., Que T., Zhang X., Li Z., Wang G., Yi L., Liu Z. (2014). Alpha-enolase as a potential cancer prognostic marker promotes cell growth, migration, and invasion in glioma. Mol. Cancer.

[126] Hsiao K.C., Shih N.Y., Fang H.L., Huang T.S., Kuo C.C., Chu P.Y., Hung Y.M., Chou S.W., Yang Y.Y., Chang G.C. (2013). Surface α-Enolase Promotes Extracellular Matrix Degradation and Tumor Metastasis and Represents a New Therapeutic Target. PLoS ONE.

[127] Principe M., Ceruti P., Shih N.Y., Chattaragada M.S., Rolla S., Conti L., Bestagno M., Zentilin L., Yang S.H., Migliorini P. (2015). Targeting of surface alpha-enolase inhibits the invasiveness of pancreatic cancer cells. Oncotarget.

[128] Principe M., Borgoni S., Cascione M., Chattaragada M.S., Ferri-Borgogno S., Capello M., Bulfamante S., Chapelle J., Di Modugno F., Defilippi P. (2017). Alpha-enolase (ENO1) controls alpha v/beta 3 integrin expression and regulates pancreatic cancer adhesion, invasion, and metastasis. J. Hematol. Oncol..

[129] Miles L.A., Dahlberg C.M., Plescia J., Felez J., Kato K., Plow E.F. (1991). Role of cell-surface lysines in plasminogen binding to cells: Identification of alpha-enolase as a candidate plasminogen receptor. Biochemistry.

[130] López-Alemany R., Longstaff C., Hawley S., Mirshahi M., Fábregas P., Jardí M., Merton E., Miles L.A., Félez J. (2003). Inhibition of cell surface mediated plasminogen activation by a monoclonal antibody against α-Enolase. Am. J. Hematol..

[131] Legler D.F., Johnson-Léger C., Wiedle G., Bron C., Imhof B.A. (2004). The αvβ3 integrin as a tumor homing ligand for lymphocytes. Eur. J. Immunol..

[132] Liu Z., Wang F., Chen X. (2008). Integrin α v β 3-targeted cancer therapy. Drug Dev. Res..

[133] Yin H., Wang L., Liu H.-L. (2018). ENO1 Overexpression in Pancreatic Cancer Patients and Its Clinical and Diagnostic Significance. Gastroenterol. Res. Pract..

[134] Ziegler Y.S., Moresco J.J., Yates J.R., Nardulli A.M. (2016). Integration of Breast Cancer Secretomes with Clinical Data Elucidates Potential Serum Markers for Disease Detection, Diagnosis, and Prognosis. PLoS ONE.

[135] Tsai S.-T., Chien I.-H., Shen W.-H., Kuo Y.-Z., Jin Y.-T., Wong T.-Y., Hsiao J.-R., Wang H.-P., Shih N.-Y., Wu L.-W. (2010). ENO1, a potential prognostic head and neck cancer marker, promotes transformation partly via chemokine CCL20 induction. Eur. J. Cancer.

[136] Liberti M.V., Locasale J.W., Biology C., Biology C. (2017). Review- The Warburg Effect: How Does it Benefit Cancer Cells ?. Trends Biochem. Sci..

[137] Zhu W., Ma L., Qian J., Xu J., Xu T., Pang L., Zhou H., Shu Y., Zhou J. (2018). The molecular mechanism and clinical significance of LDHA in HER2-mediated progression of gastric cancer. Am. J. Transl. Res..

[138] Rong Y., Wu W., Ni X., Kuang T., Jin D., Wang D., Lou W. (2013). Lactate dehydrogenase A is overexpressed in pancreatic cancer and promotes the growth of pancreatic cancer cells. Tumor Biol..

[139] Liu Y., Guo J.Z., Liu Y., Wang K., Ding W., Wang H., Liu X., Zhou S., Lu X.C., Yang H.B. (2018). Nuclear lactate dehydrogenase A senses ROS to produce α-hydroxybutyrate for HPV-induced cervical tumor growth. Nat. Commun..

[140] Feng Y., Xiong Y., Qiao T., Li X., Jia L., Han Y. (2018). Lactate dehydrogenase A: A key player in carcinogenesis and potential target in cancer therapy. Cancer Med..

[141] Yu X., Li S. (2017). Non-metabolic functions of glycolytic enzymes in tumorigenesis. Oncogene.

[142] Steeg P.S., Kopper L., Thorgeirsson U.P., Talmadge E., Liotta L.A., Sobep M.E. (1988). Evidence for a Novel Gene Associated With Low Tumor Metastatic Potential. J. Natl. Cancer Inst..

[143] Ohshio G., Imamura T., Okada N., Suwa H., Yamaki K., Imamura M., Ogasahara K., Tsukayama C., Yamabe H. (2008). Immunohistochemical expression of nm23 gene product, nucleotide diphosphate kinase, in pancreatic neoplasms. Int. J. Gastrointest. Cancer.

[144] Nakamori S., Ishikawa O., Ohigashi H., Imaoka S., Sasaki Y., Kameyama M., Kabuto T., Furukawa H., Iwanakga T., Kimura N. (1993). Clinicopathological features and prognostic significance of nucleoside diphosphate kinase/nm23 gene product in human pancreatic exocrine neoplasms. Int. J. Pancreatol..

[145] Takadate T., Onogawa T., Fujii K., Motoi F., Mikami S., Fukuda T., Kihara M., Suzuki T., Takemura T., Minowa T. (2012). NM23/nucleoside diphosphate kinase-A as a potent prognostic marker in invasive pancreatic ductal carcinoma identified by proteomic analysis of laser micro-dissected formalin-fixed paraffin-embedded tissue. Clin. Proteom..

[146] Liu L., Li M., Zhang C., Zhang J., Li G., Zhang Z., He X., Fan M. (2018). Prognostic value and clinicopathologic significance of nm23 in various cancers: A systematic review and meta-analysis. Int. J. Surg..

[147] Royds J.A., Cross S.S., Silcocks P.B., Scholefield J.H., Rees R.C., Stephenson T.J. (1994). Nm23 ‘anti-metastatic’ gene product expression in colorectal carcinoma. J. Pathol..

[148] Orozco C.A., Martinez-Bosch N., Guerrero P.E., Vinaixa J., Dalotto-Moreno T., Iglesias M., Moreno M., Djurec M., Poirier F., Gabius H.-J. (2018). Targeting galectin-1 inhibits pancreatic cancer progression by modulating tumor–stroma crosstalk. Proc. Natl. Acad. Sci. USA.

[149] Zhang P.F., Li K.S., Shen Y.H., Gao P.T., Dong Z.R., Cai J.B., Zhang C., Huang X.Y., Tian M.X., Hu Z.Q. (2016). Galectin-1 induces hepatocellular carcinoma EMT and sorafenib resistance by activating FAK/PI3K/AKT signaling. Cell Death Dis..

[150] Van Woensel M., Mathivet T., Wauthoz N., Rosière R., Garg A.D., Agostinis P., Mathieu V., Kiss R., Lefranc F., Boon L. (2017). Sensitization of glioblastoma tumor micro-environment to chemo- and immunotherapy by Galectin-1 intranasal knock-down strategy. Sci. Rep..

[151] Yeh C.-C., Hsu C.-H., Shao Y.-Y., Ho W.-C., Tsai M.-H., Feng W.-C., Chow L.-P. (2015). Integrated Stable Isotope Labeling by Amino Acids in Cell Culture (SILAC) and Isobaric Tags for Relative and Absolute Quantitation (iTRAQ) Quantitative Proteomic Analysis Identifies Galectin-1 as a Potential Biomarker for Predicting Sorafenib Resistance i. Mol. Cell. Proteom..

[152] Su Y.C., Davuluri G.V.N., Chen C.H., Shiau D.C., Chen C.C., Chen C.L., Lin Y.S., Chang C.P. (2016). Galectin-1-induced autophagy facilitates cisplatin resistance of hepatocellular carcinoma. PLoS ONE.

[153] Chung L.Y., Tang S.J., Sun G.H., Chou T.Y., Yeh T.S., Yu S.L., Sun K.H. (2012). Galectin-1 promotes lung cancer progression and chemoresistance by upregulating p38 MAPK, ERK, and cyclooxygenase-2. Clin. Cancer Res..

[154] Zhang P., Zhang P., Shi B., Zhou M., Jiang H., Zhang H., Pan X., Gao H., Sun H., Li Z. (2014). Galectin-1 overexpression promotes progression and chemoresistance to cisplatin in epithelial ovarian cancer. Cell Death Dis..

[155] Mathieu V., Le Mercier M., De Neve N., Sauvage S., Gras T., Roland I., Lefranc F., Kiss R. (2007). Galectin-1 knockdown increases sensitivity to temozolomide in a B16F10 mouse metastatic melanoma model. J. Investig. Dermatol..

[156] Lykken J.M., Horikawa M., Minard-Colin V., Kamata M., Miyagaki T., Poe J.C., Tedder T.F. (2016). Galectin-1 drives lymphoma CD20 immunotherapy resistance: Validation of a preclinical system to identify resistance mechanisms. Blood.

[157] Cui G., Cui M., Li Y., Liang Y., Li W., Guo H., Zhao S. (2015). Galectin-3 knockdown increases gefitinib sensitivity to the inhibition of EGFR endocytosis in gefitinib-insensitive esophageal squamous cancer cells. Med. Oncol..

[158] Mirandola L., Yu Y., Cannon M.J., Jenkins M.R., Rahman R.L., Nguyen D.D., Grizzi F., Cobos E., Figueroa J.A., Chiriva-Internati M. (2014). Galectin-3 inhibition suppresses drug resistance, motility, invasion and angiogenic potential in ovarian cancer. Gynecol. Oncol..

[159] Streetly M.J., Maharaj L., Joel S., Schey S.A., Gribben J.G., Cotter F.E. (2010). GCS-100, a novel galectin-3 antagonist, modulates MCL-1, NOXA, and cell cycle to induce myeloma cell death. Blood.

[160] Mazurek N., Byrd J.C., Sun Y., Hafley M., Ramirez K., Burks J., Bresalier R.S. (2012). Cell-surface galectin-3 confers resistance to TRAIL by impeding trafficking of death receptors in metastatic colon adenocarcinoma cells. Cell Death Differ..

[161] Kyu J.C., Yu J.P., Min J.L., Jin H.K., Ha J., Choe W., Sung S.K. (2007). Overexpressed cyclophilin A in cancer cells renders resistance to hypoxia- and cisplatin-induced cell death. Cancer Res..

[162] Zhu X., Miao X., Wu Y., Li C., Guo Y., Liu Y., Chen Y., Lu X., Wang Y., He S. (2015). ENO1 promotes tumor proliferation and cell adhesion mediated drug resistance (CAM-DR) in Non-Hodgkin’s Lymphomas. Exp. Cell Res..

[163] Maiso P., Huynh D., Moschetta M., Sacco A., Aljawai Y., Mishima Y., Asara J.M., Roccaro A.M., Kimmelman A.C., Ghobrial I.M. (2015). Metabolic Signature Identifies Novel Targets for Drug Resistance in Multiple Myeloma. Cancer Res..

[164] Zeng H.Z., Qu Y.Q., Zhang W.J., Xiu B., Deng A.M., Liang A. (2011). Bin Proteomic analysis identified DJ-1 as a cisplatin resistant marker in non-small cell lung cancer. Int. J. Mol. Sci..

[165] Sagulenko V., Muth D., Sagulenko E., Paffhausen T., Schwab M., Westermann F. (2008). Cathepsin D protects human neuroblastoma cells from doxorubicin-induced cell death. Carcinogenesis.

[166] Bai D., Ueno L., Vogt P. (2009). Akt-mediated regulation of NFkB for the oncogenicity of PI3K and Akt. Int. J. Cancer.

[167] Fresno Vara J.Á., Casado E., de Castro J., Cejas P., Belda-Iniesta C., González-Barón M. (2004). P13K/Akt signalling pathway and cancer. Cancer Treat. Rev..

[168] Gry M., Rimini R., Strömberg S., Asplund A., Pontén F., Uhlén M., Nilsson P. (2009). Correlations between RNA and protein expression profiles in 23 human cell lines. BMC Genom..

[169] Pillai R.S., Bhattacharyya S.N., Filipowicz W. (2007). Repression of protein synthesis by miRNAs: How many mechanisms?. Trends Cell Biol..

[170] Hausser J., Zavolan M. (2014). Identification and consequences of miRNA-target interactions-beyond repression of gene expression. Nat. Rev. Genet..

[171] Raphael B.J., Hruban R.H., Aguirre A.J., Moffitt R.A., Yeh J.J., Stewart C., Robertson A.G., Cherniack A.D., Gupta M., Getz G. (2017). Integrated Genomic Characterization of Pancreatic Ductal Adenocarcinoma. Cancer Cell.

[172] Felix T.F., Lopez Lapa R.M., De Carvalho M., Bertoni N., Tokar T., Oliveira R.A., Rodrigues M.A.M., Hasimoto C.N., Oliveira W.K., Pelafsky L. (2019). MicroRNA modulated networks of adaptive and innate immune response in pancreatic ductal adenocarcinoma. PLoS ONE.

[173] Slater E.P., Fendrich V., Strauch K., Rospleszcz S., Ramaswamy A., Matthäi E., Chaloupka B., Gress T.M., Langer P., Bartsch D.K. (2013). LCN2 and TIMP1 as potential serum markers for the early detection of familial pancreatic cancer. Transl. Oncol..

[174] Bartsch D., Gercke N., Strauch K., Wieboldt R., Matthäi E., Wagner V., Rospleszcz S., Schäfer A., Franke F., Mintziras I. (2018). The Combination of MiRNA-196b, LCN2, and TIMP1 is a Potential Set of Circulating Biomarkers for Screening Individuals at Risk for Familial Pancreatic Cancer. J. Clin. Med..

[175] Yu S., Li Y., Liao Z., Wang Z., Wang Z., Li Y., Qian L., Zhao J., Zong H., Kang B. (2020). Plasma extracellular vesicle long RNA profiling identifies a diagnostic signature for the detection of pancreatic ductal adenocarcinoma. Gut.

[176] Xu Q., Li P., Chen X., Zong L., Jiang Z., Nan L., Lei J., Duan W., Zhang D., Li X. (2015). MiR-221/222 induces pancreatic cancer progression through the regulation of matrix metalloproteinases. Oncotarget.

[177] Moriyama T., Ohuchida K., Mizumoto K., Yu J., Sato N., Nabae T., Takahata S., Toma H., Nagai E., Tanaka M. (2009). MicroRNA-21 modulates biological functions of pancreatic cancer cells including their proliferation, invasion, and chemoresistance. Mol. Cancer Ther..

[178] Freire P.P., Fernandez G.J., Moraes D.D., Cury S.S., Pai-Silva M.D., Pintor P., Rogatto S.R., Carvalho R.F. (2020). The expression landscape of cachexia-inducing factors in human cancers. J. Cachexia Sarcopenia Muscle.

[179] Robinson J.L., Feizi A., Uhlén M., Nielsen J. (2019). A Systematic Investigation of the Malignant Functions and Diagnostic Potential of the Cancer Secretome. Cell Rep..

[180] Yuan F., Zhang Y.H., Wan S., Wang S., Kong X.Y. (2015). Mining for candidate genes related to pancreatic cancer using protein-protein interactions and a shortest path approach. Biomed Res. Int..

[181] Vareed S.K., Bhat V.B., Thompson C., Vasu V.T., Fermin D., Choi H., Creighton C.J., Gayatri S., Lan L., Putluri N. (2011). Metabolites of purine nucleoside Phosphorylase (NP) in serum have the potential to delineate Pancreatic Adenocarcinoma. PLoS ONE.

[182] He X., Zheng Z., Li J., Ben Q., Liu J., Zhang J., Ji J., Yu B., Chen X., Su L. (2012). DJ-1 promotes invasion and metastasis of pancreatic cancer cells by activating SRC/ERK/uPA. Carcinogenesis.

[183] Maurer H.C., Holmstrom S.R., He J., Laise P., Su T., Ahmed A., Hibshoosh H., Chabot J.A., Oberstein P.E., Sepulveda A.R. (2019). Experimental microdissection enables functional harmonisation of pancreatic cancer subtypes. Gut.

[184] Liberati A., Altman D.G., Tetzlaff J., Mulrow C., Gøtzsche P.C., Ioannidis J.P.A., Clarke M., Devereaux P.J., Kleijnen J., Moher D. (2009). The PRISMA statement for reporting systematic reviews and meta-analyses of studies that evaluate health care interventions: Explanation and elaboration. Ann. Intern. Med..

[185] Metsalu T., Vilo J. (2015). ClustVis: A web tool for visualizing clustering of multivariate data using Principal Component Analysis and heatmap. Nucleic Acids Res..

[186] Starruß J., De Back W., Brusch L., Deutsch A. (2014). Morpheus: A user-friendly modeling environment for multiscale and multicellular systems biology. Bioinformatics.

