# Supplementary material for: An Integrated Meta-Analysis of Secretome and Proteome Identify Potential Biomarkers of Pancreatic Ductal Adenocarcinoma
Source: Cancers (Basel). 2020 Mar 18;12(3):716. doi: 10.3390/cancers12030716 (PMC7140071; doi:10.3390/cancers12030716)

**Supplementary Figure 1**. Expression profile of 39 secretome genes in normal and tumor tissues from GEPIA (Gene Expression web-based Profiling Analysis) and GTEx (Genotype-Tissue Expression), respectively. A) Schematic representation of the expression profile of 39 mRNAs translated into proteins identified as secreted in 10 different tumor types. The differential expression levels from tumor tissue versus combined normal TCGA and GTEx data were calculated using the web-based Gene Expression Profiling Analysis tool (GEPIA, http://gepia.cancerpku.cn/) [105]. Positively or negatively regulated genes (red and green, respectively) with absolute values of expression change (fold-change) > 1.0 and q-value < 0.01 (ANOVA). The number at the bottom of each column represents the number of times that a single gene is upregulated (red square) across tumor types, while the number on the right side of each row represents the number of upregulated genes in a single tumor type (red square). B) Heatmap of the gene expression profile in normal tissues from GTEx encoding 39 proteins, which were identified as secreted in PAAD. TPM values ​​are represented by color intensity; dark blue indicates transcripts per million (TPM) values > 1.0 e+3 and light blue indicates TPM values ​​<1.0 e+3. Arrow indicates pancreatic tissue. PDAC: Pancreatic ductal adenocarcinoma; GC: gastric carcinoma; COAD: Colon adenocarcinoma; HCC: Hepatocellular carcinoma; Lung SCC: Lung squamous cell carcinoma; BC: Breast cancer; HNSCC: Head and neck squamous cell carcinoma; ESCC: Esophageal squamous cell carcinoma; Lung AD: Lung adenocarcinoma; AML: Acute myeloid leukemia.

**
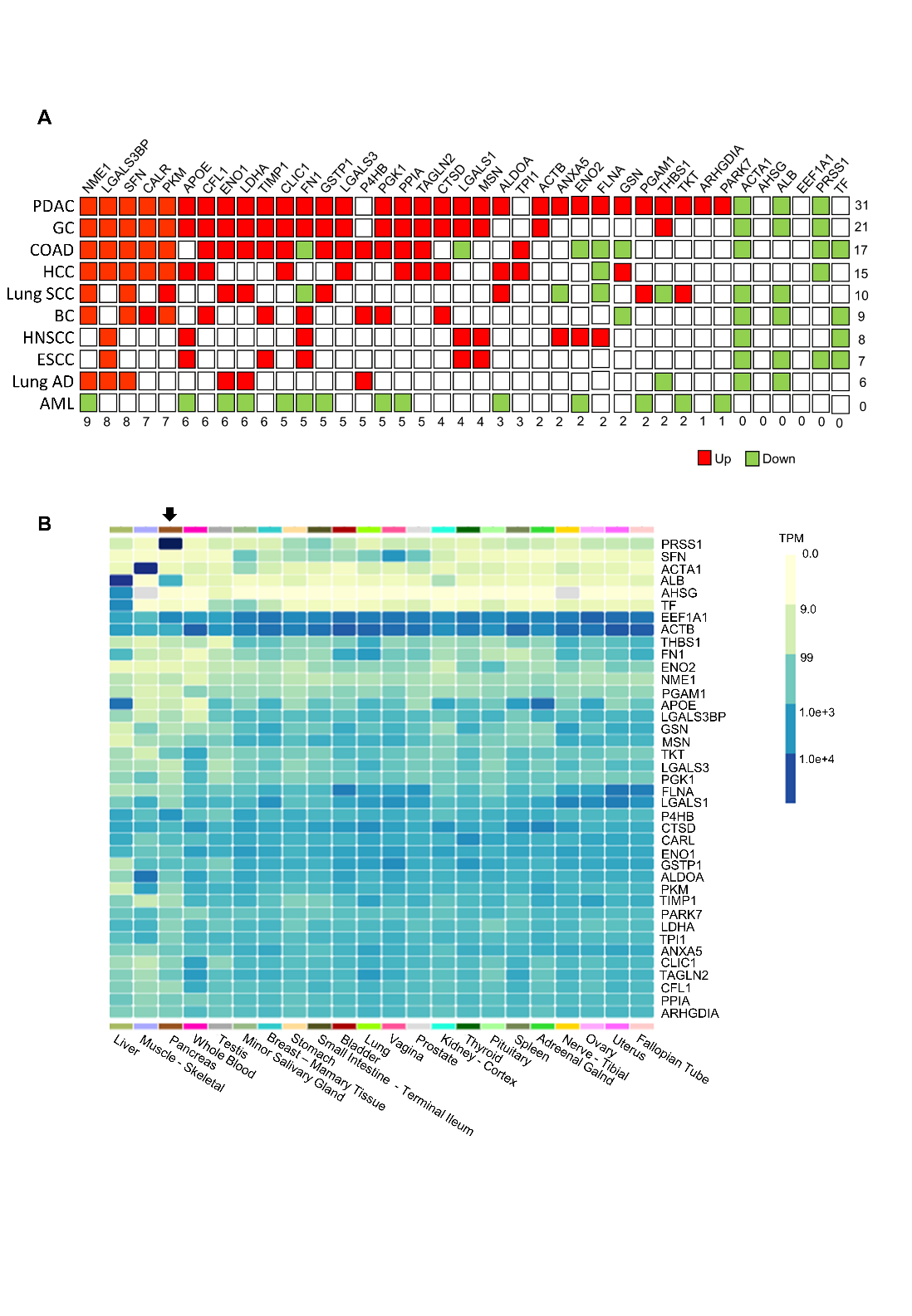
**

**Supplementary Figure 2.** Principal component analysis (PCA) of 39 secretome genes in 10 different tumor types from TCGA compared to their respective normal tissues. The dots represent the expression profile for the secretome genes in each cancer type. PDAC: Pancreatic ductal adenocarcinoma; GC: gastric carcinoma; COAD: Colon adenocarcinoma; HCC: Hepatocellular carcinoma; Lung SCC: Lung squamous cell carcinoma; BC: Breast cancer; HNSCC: Head and neck squamous cell carcinoma; ESCC: Esophageal squamous cell carcinoma; Lung AD: Lung adenocarcinoma; AML: Acute myeloid leukemia.


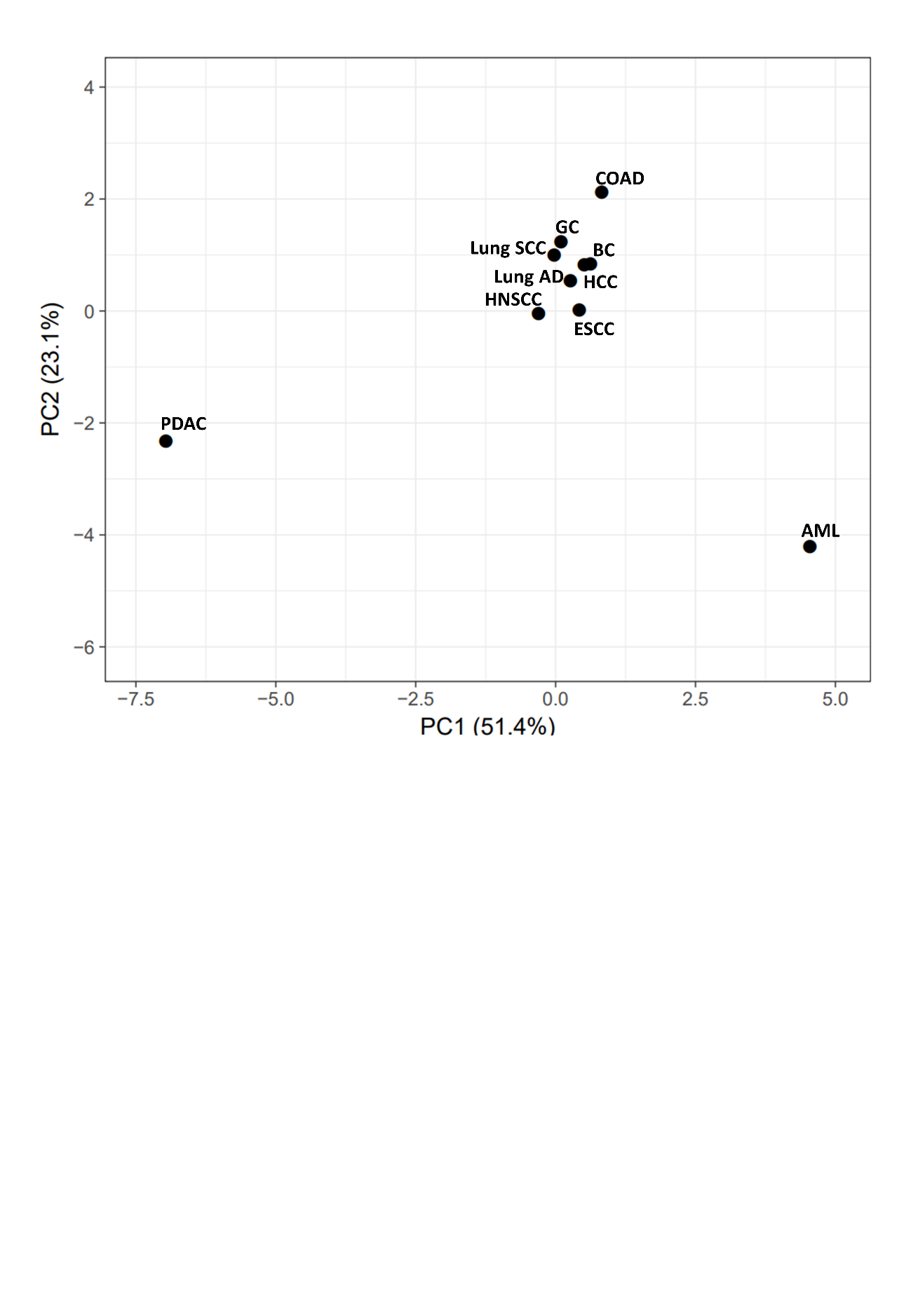


**Supplementary Figure 3.** The expression profile of 39 secretome genes by PAAD stratifies patients into low- and high-risk groups. Heatmaps represent the nonhierarchical cluster analysis of the gene expression profile in 10 different tumor types from TCGA. Cancer patients were stratified into high- and low-risk groups, indicated below each heatmap as red and green bars, respectively. The risk groups were maximized based on the Prognostic Index evaluated by gene expression values multiplied by beta coefficients. HNSCC: Head and neck squamous cell carcinoma; ESCC: Esophageal squamous cell carcinoma; GC: gastric carcinoma; HCC: Hepatocellular carcinoma; Lung AD: Lung adenocarcinoma; Lung SCC: Lung squamous cell carcinoma; COAD: Colon adenocarcinoma; PDAC: Pancreatic ductal adenocarcinoma; AML: Acute myeloid leukemia; BC: Breast cancer.

**
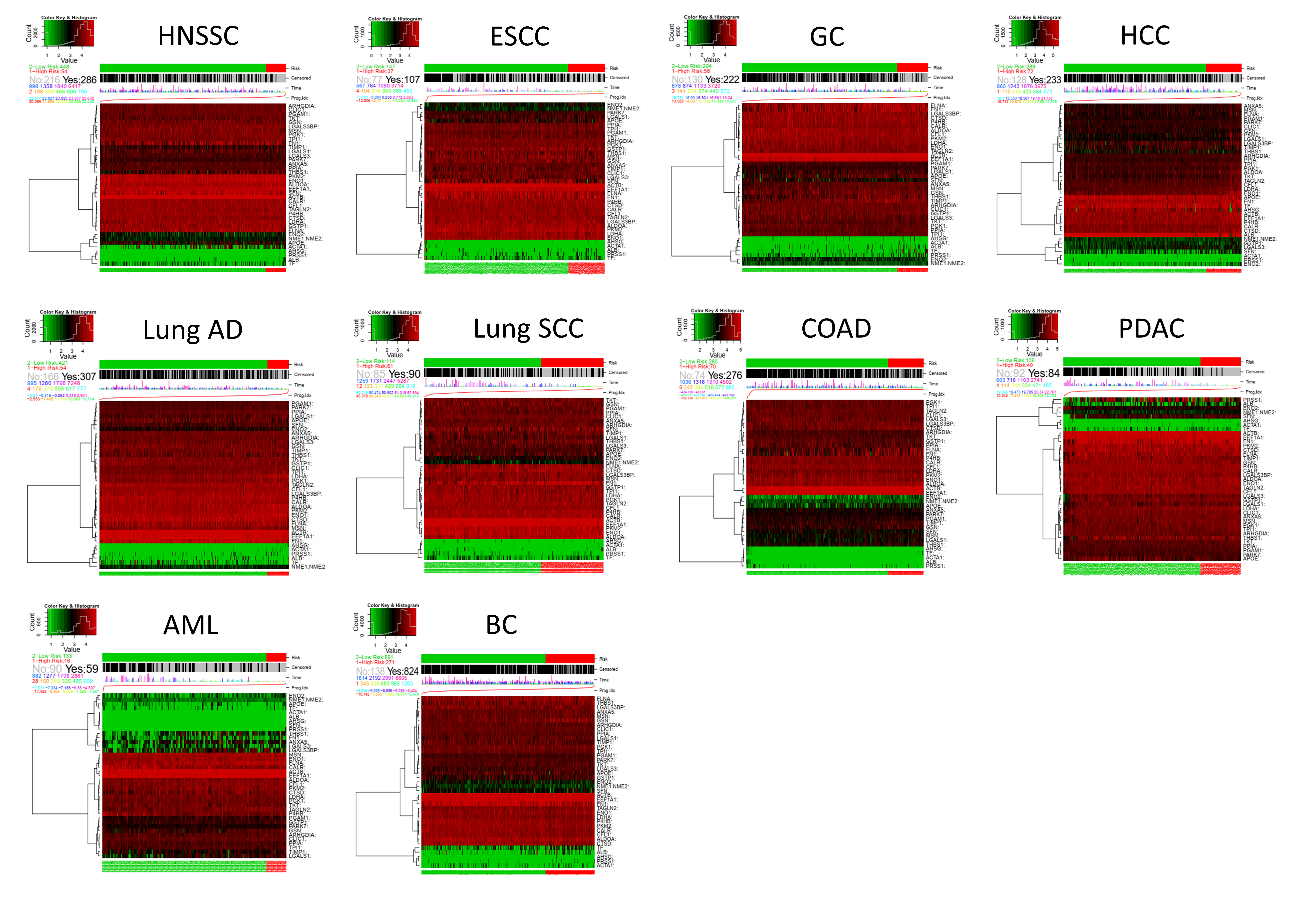
**

**Supplementary Figure 4.** Validation of secreted protein expression in normal and PAAD tissues using immunohistochemical staining available at the Human Protein Atlas database (https://www.proteinatlas.org/). These proteins were selected based on their significant increased gene expression as identified using the GEPIA tool (<http://gepia.cancerpku.cn/>).
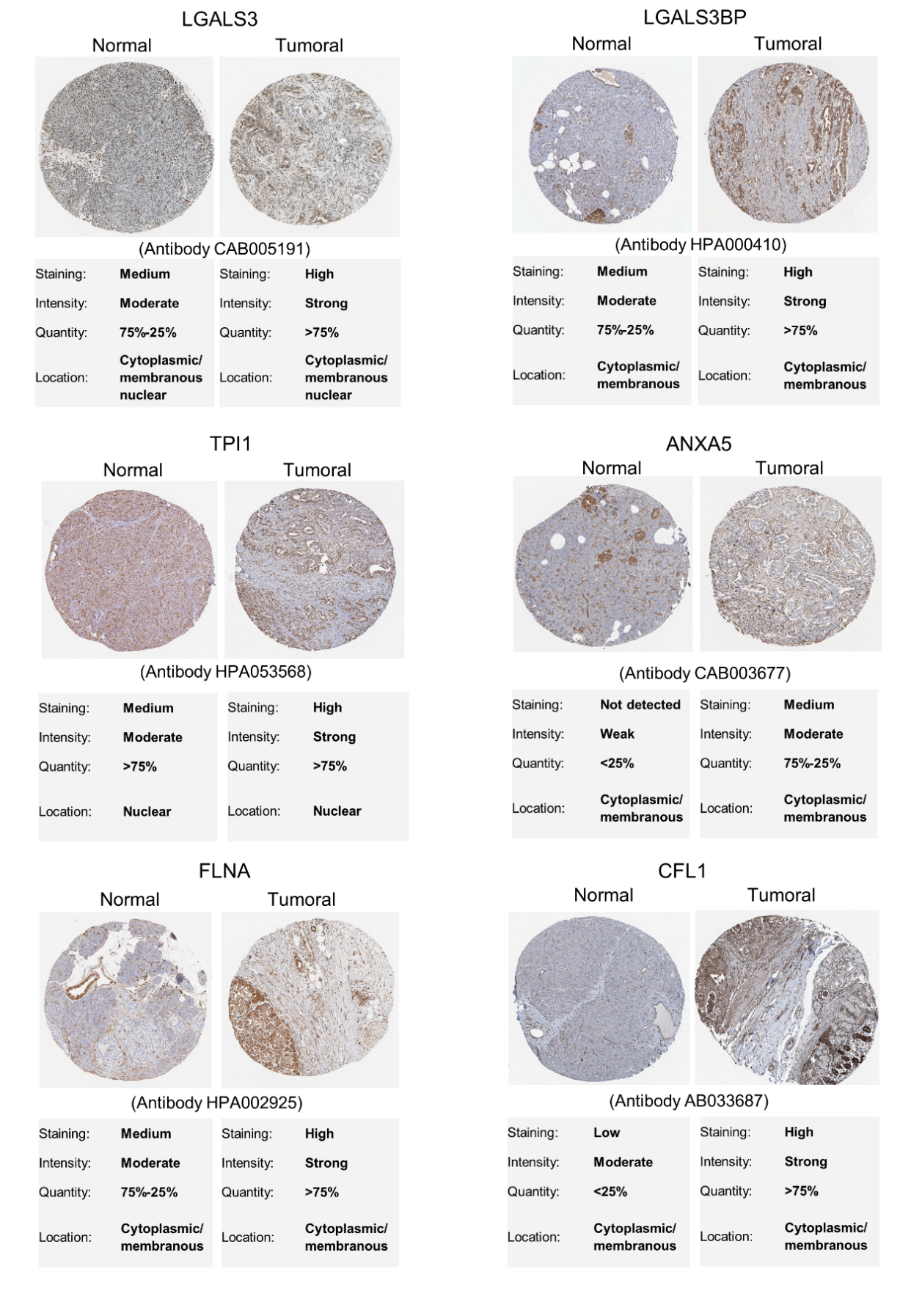

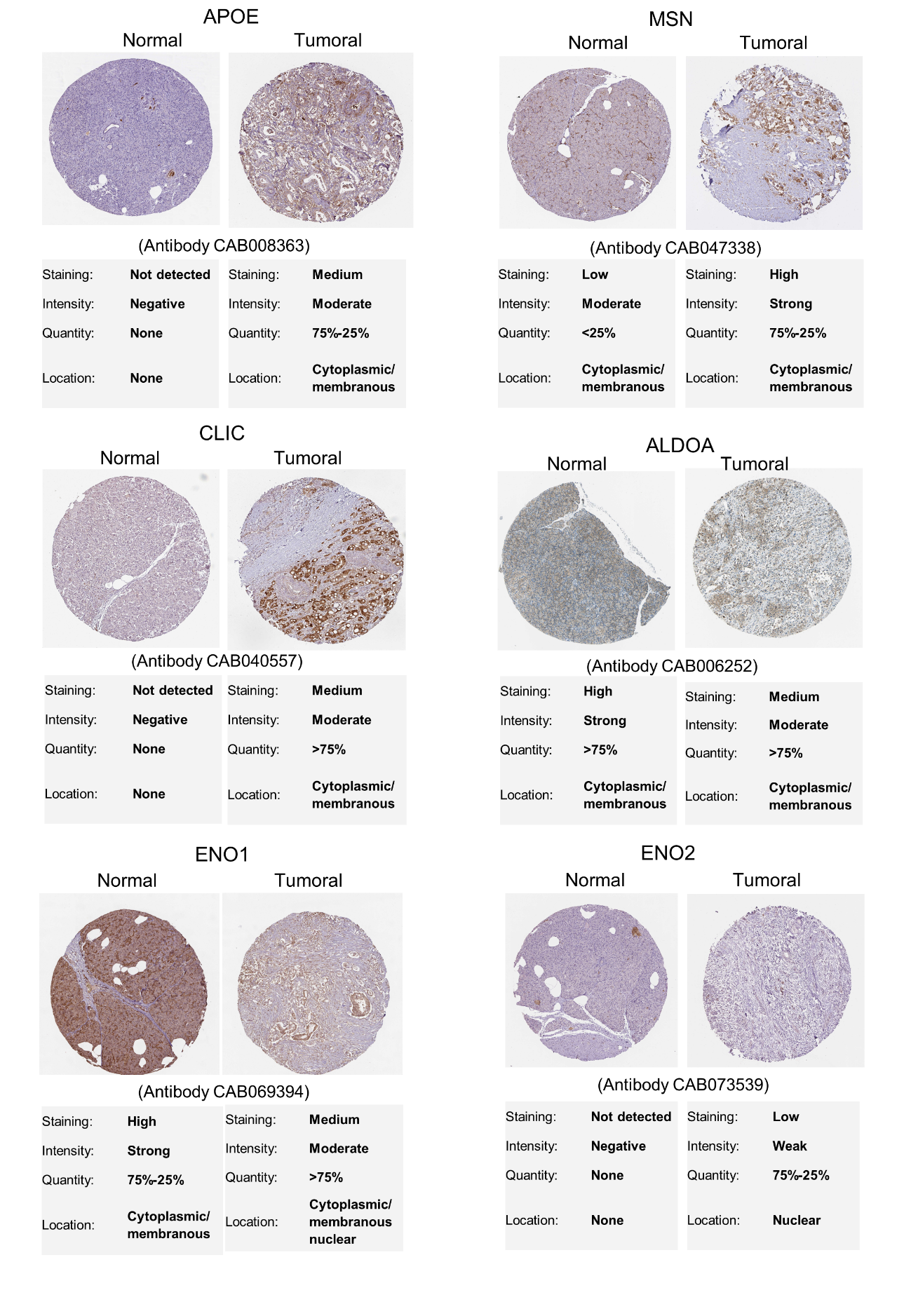

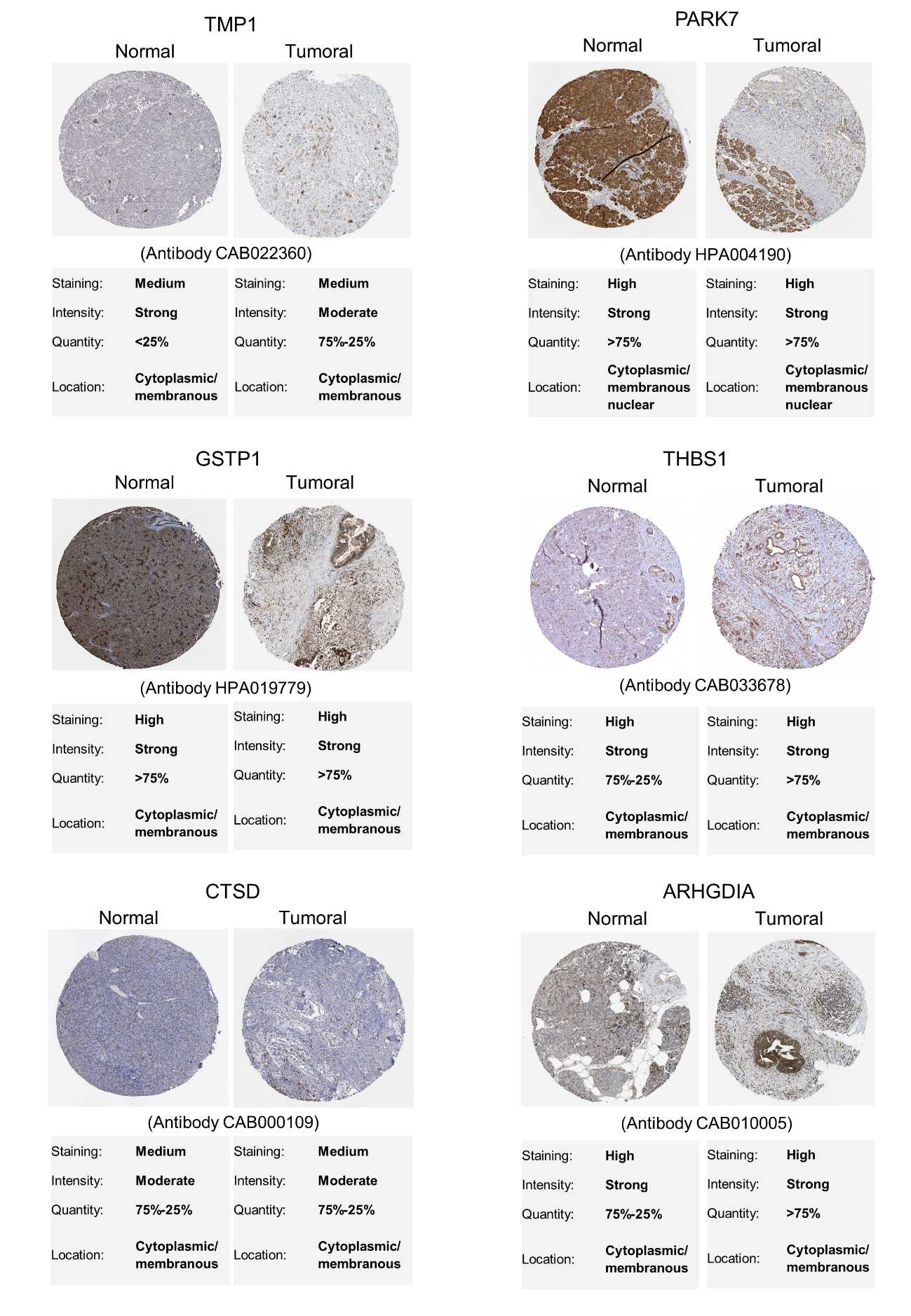

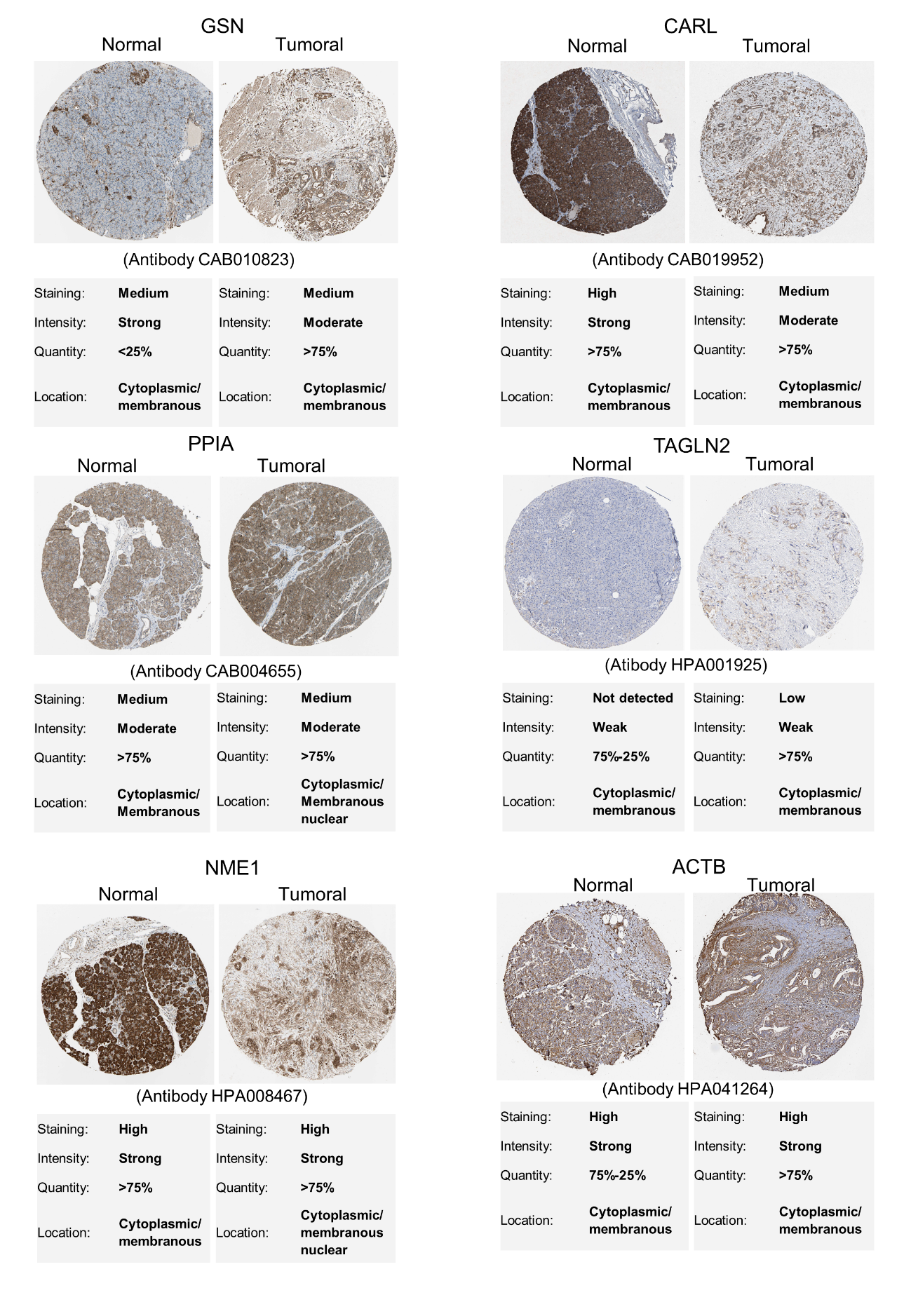

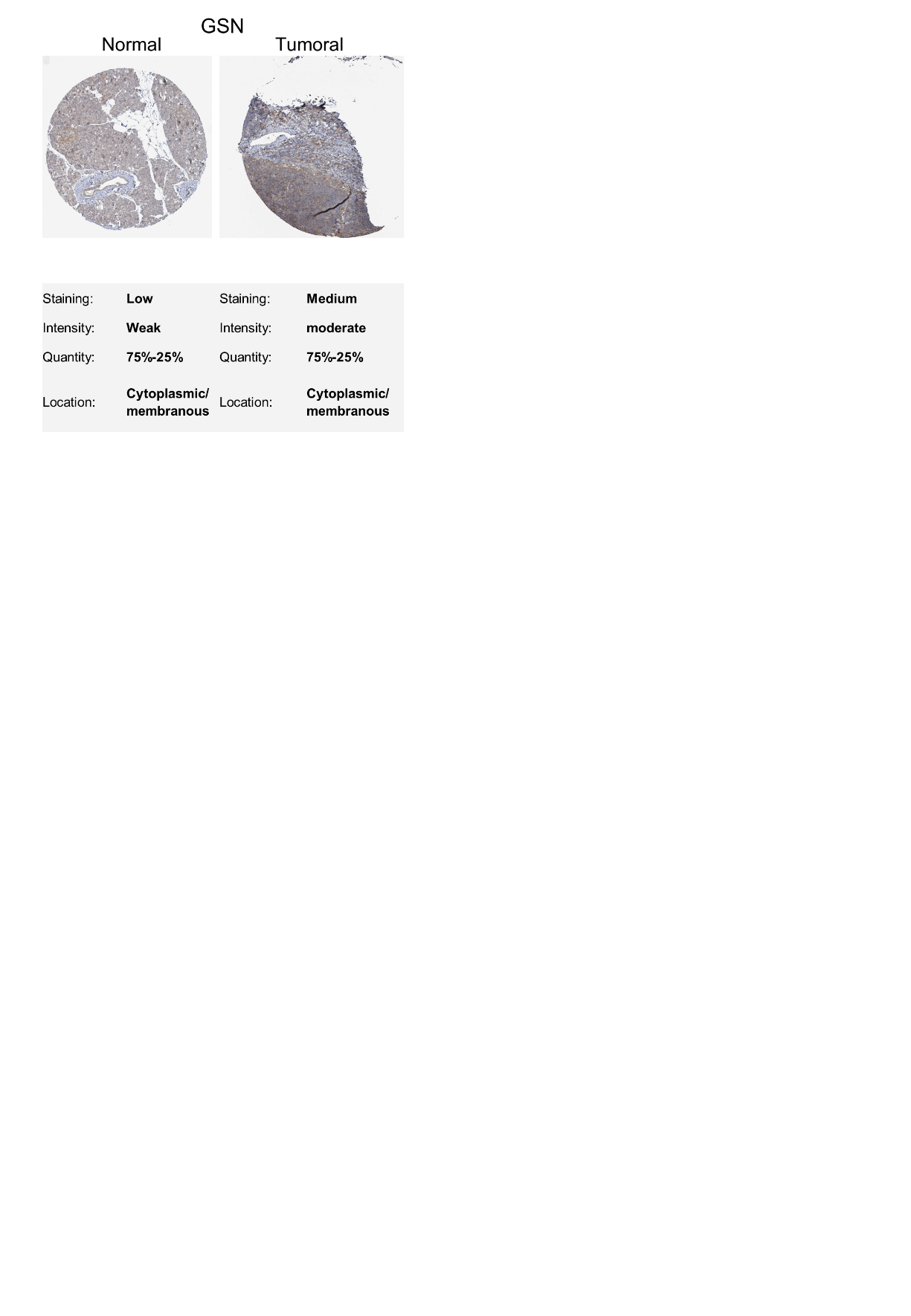

Supplement: Supplementary file 1 [file cancers-12-00716-s001.zip › Supplementary Figures.docx]
