# Supplementary material for: An Integrated Meta-Analysis of Secretome and Proteome Identify Potential Biomarkers of Pancreatic Ductal Adenocarcinoma
Source: Cancers (Basel). 2020 Mar 18;12(3):716. doi: 10.3390/cancers12030716 (PMC7140071; doi:10.3390/cancers12030716)
Supplement: Supplementary file 1 [file cancers-12-00716-s001.zip › ST 1.docx]

**Supplementary Table 1.** Total number of proteins identified in proteomic studies of pancreatic cancer.

|  | *Proteome* | *Secretome* |
| --- | --- | --- |
| Total unique proteins | 517 | 782 |
| [in ≥ two studies] | [132] | [156] |
| Number of proteins identified in | | |
| One study | 385 | 626 |
| Two studies | 67 | 101 |
| Three studies | 27 | 35 |
| Four studies | 19 | 11 |
| Five studies | 9 | 5 |
| Six studies | 7 | 3 |
| Seven studies | 0 | 1 |
| Eight studies | 2 | 0 |
| Nine studies | 0 | 0 |
| Ten studies | 1 | 0 |
