# Supplementary material for: An Integrated Meta-Analysis of Secretome and Proteome Identify Potential Biomarkers of Pancreatic Ductal Adenocarcinoma
Source: Cancers (Basel). 2020 Mar 18;12(3):716. doi: 10.3390/cancers12030716 (PMC7140071; doi:10.3390/cancers12030716)
Supplement: Supplementary file 1 [file cancers-12-00716-s001.zip › ST 7.docx]

Supplementary Table 7. Expression of 39 secreted proteins based on samples from four different PDAC data sets predicts poor overall survival.

| **PDAC**  **Study** | Samples | Censored | Survival Data | High-Risk (%) | Low-Risk (%) | p-Risk Group | Overall C-index | Log Rank Test |
| --- | --- | --- | --- | --- | --- | --- | --- | --- |
| TCGA | 176 | 84 | days | 22.7 | 77.3 | > 0.000001 | 74.7 | > 0.000001 |
| PACA-AU-ICGC | 189 | 79 | days | 32.28 | 67.72 | > 0.000001 | 72.6 | > 0.000001 |
| GSE28735 | 90 | 27 | months | 42.86 | 57,14 | > 0.000001 | 84.5 | > 0.000001 |
| GSE21501 | 132 | 36 | months | 11.76 | 88.76 | > 0.000001 | 76.7 | > 0.000001 |
