# Supplementary material for: An Integrated Meta-Analysis of Secretome and Proteome Identify Potential Biomarkers of Pancreatic Ductal Adenocarcinoma
Source: Cancers (Basel). 2020 Mar 18;12(3):716. doi: 10.3390/cancers12030716 (PMC7140071; doi:10.3390/cancers12030716)
Supplement: Supplementary file 1 [file cancers-12-00716-s001.zip › ST 9.docx]

Supplementary Table 9. The expression of 39 genes coding for secreted proteins based on The Cancer Genome Atlas (TCGA) tumor samples predicts poor overall survival.

| **TCGA Study** | Samples | Censored | Survival Data | High-Risk (%) | Low-Risk (%) | p-Risk Group | Overall C-index | Log Rank Test |
| --- | --- | --- | --- | --- | --- | --- | --- | --- |
| HNSCC | 502 | 286 | days | 10.8 | 89.2 | > 0.00001 | 65.8 | > 0.00001 |
| ESCC | 184 | 107 | days | 20.1 | 79.9 | > 0.00001 | 74.8 | > 0.00001 |
| GC | 352 | 222 | days | 16.5 | 83.5 | > 0.00001 | 67.8 | > 0.00001 |
| HCC | 361 | 233 | days | 19.9 | 80.1 | > 0.00001 | 71.1 | > 0.00001 |
| Lung AD | 475 | 307 | days | 11.4 | 88.6 | > 0.00001 | 68.4 | 0 |
| Lung SCC | 175 | 90 | days | 34.9 | 65.1 | > 0.00001 | 72.9 | > 0.00001 |
| COAD | 350 | 276 | days | 20 | 80 | > 0.00001 | 70.8 | > 0.00001 |
| AML | 149 | 59 | days | 89.3 | 10.7 | > 0.00001 | 69.1 | > 0.00001 |
| BC | 962 | 824 | days | 28.2 | 71.8 | > 0.00001 | 74.8 | 0 |
| PDAC | 176 | 84 | days | 22.7 | 77.3 | > 0.00001 | 74.7 | > 0.00001 |
