# Supplementary figures and images for: An Integrated Meta-Analysis of Secretome and Proteome Identify Potential Biomarkers of Pancreatic Ductal Adenocarcinoma
Source: Cancers (Basel). 2020 Mar 18;12(3):716. doi: 10.3390/cancers12030716 (PMC7140071; doi:10.3390/cancers12030716)

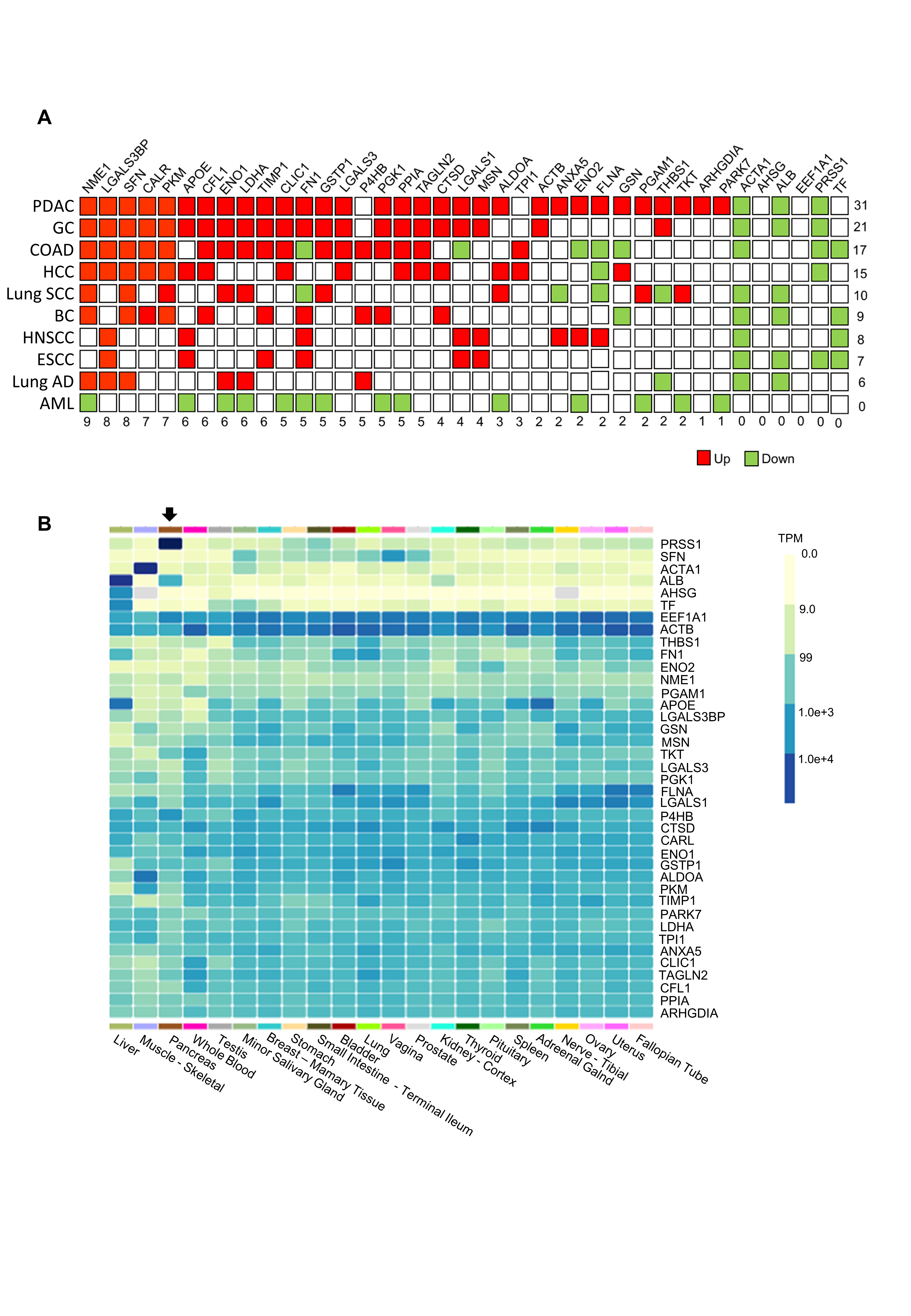

Supplement: Supplementary file 1 [file cancers-12-00716-s001.zip › Supplementary Figure 1.tif]

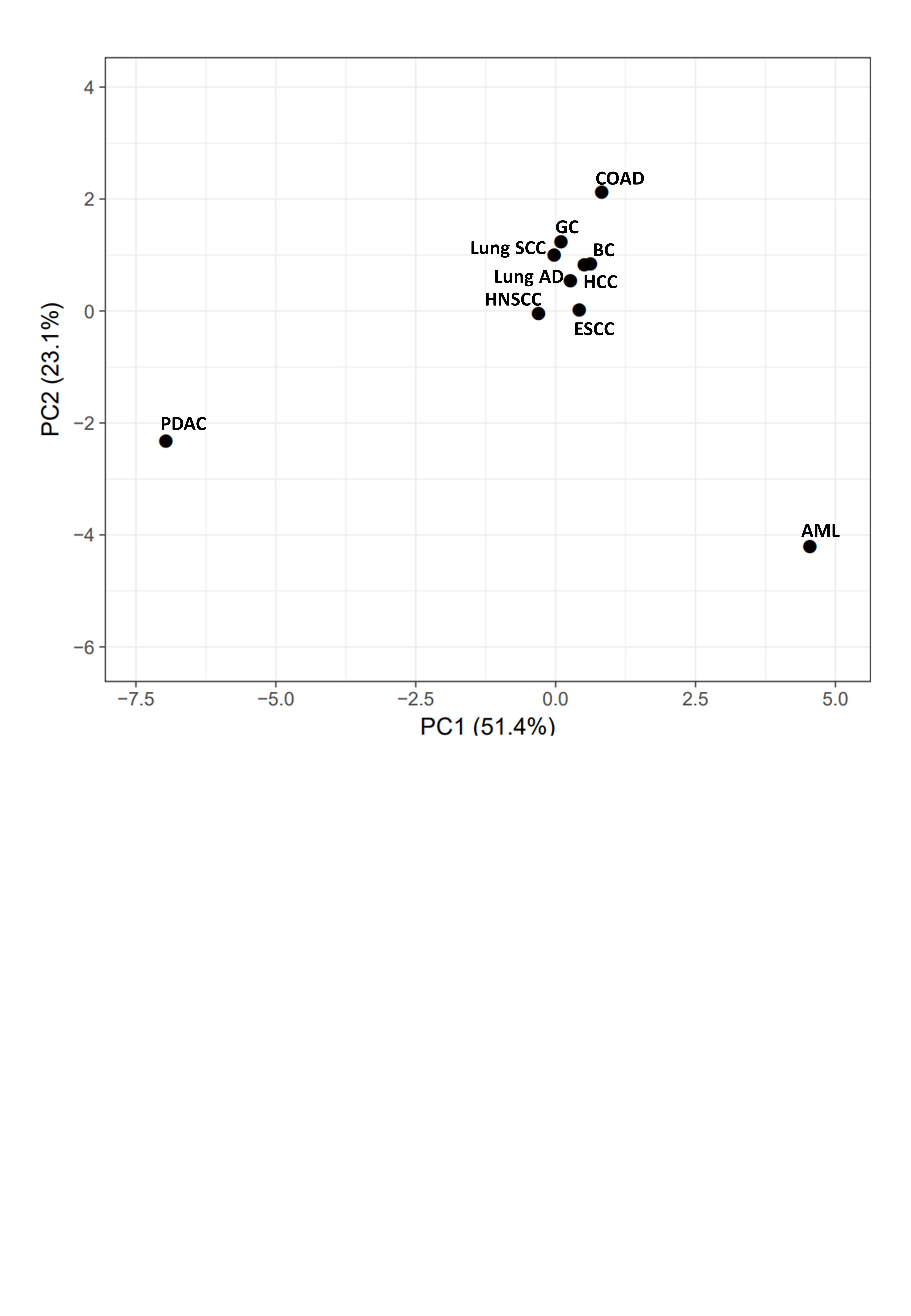

Supplement: Supplementary file 1 [file cancers-12-00716-s001.zip › Supplementary Figure 2.tif]

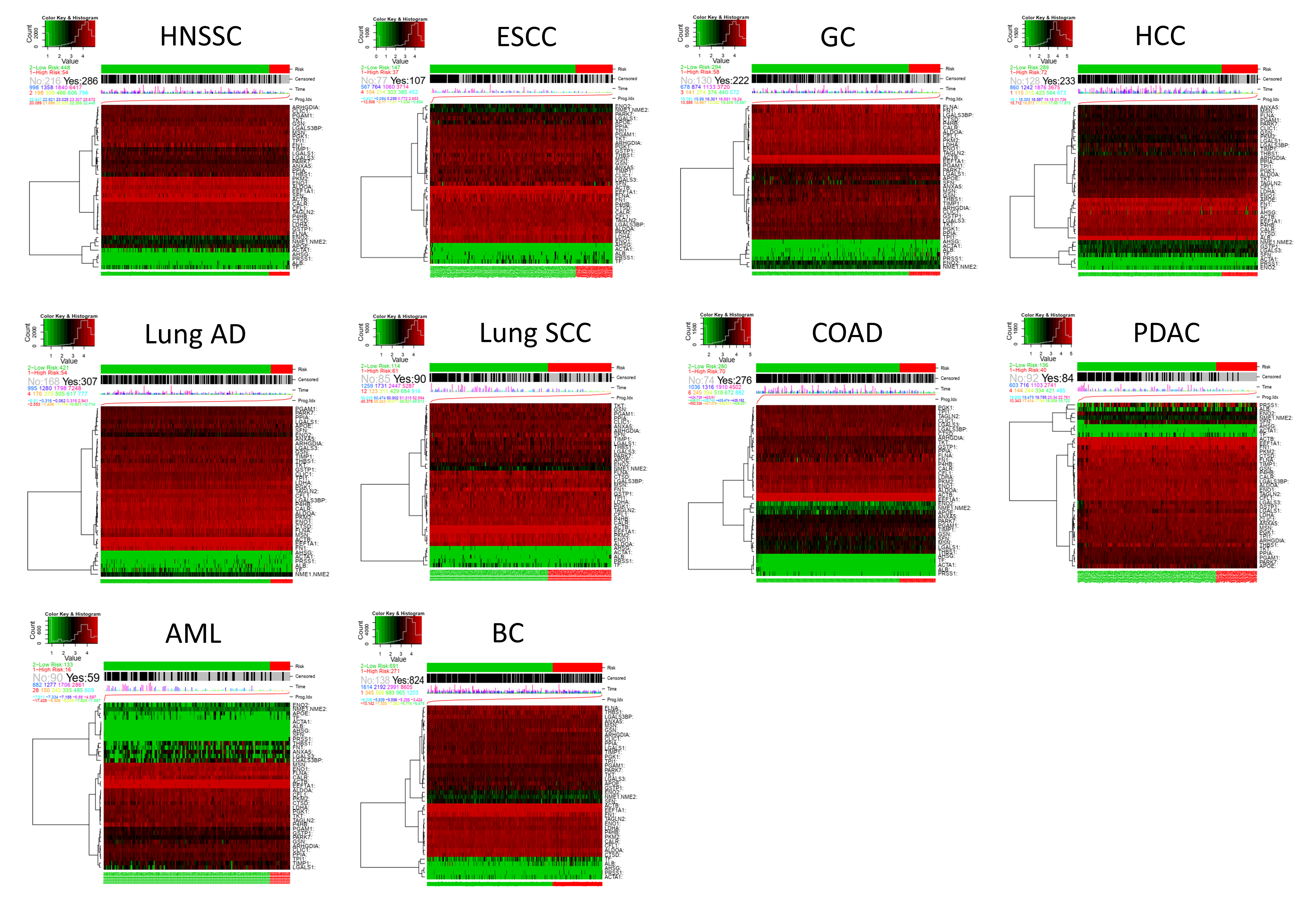

Supplement: Supplementary file 1 [file cancers-12-00716-s001.zip › Supplementary Figure 3.tif]

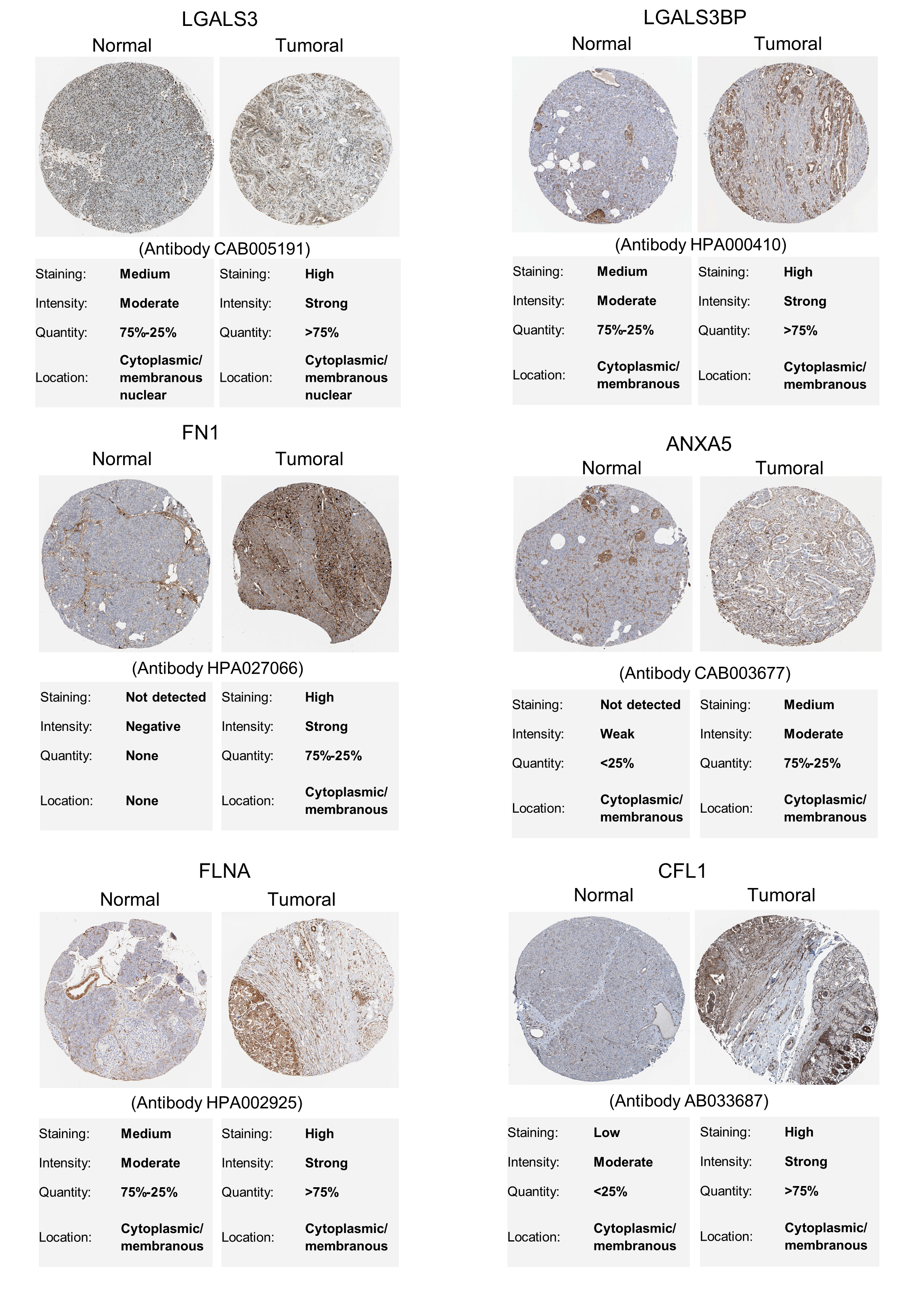

Supplement: Supplementary file 1 [file cancers-12-00716-s001.zip › Supplementary Figure 4 a.TIF]

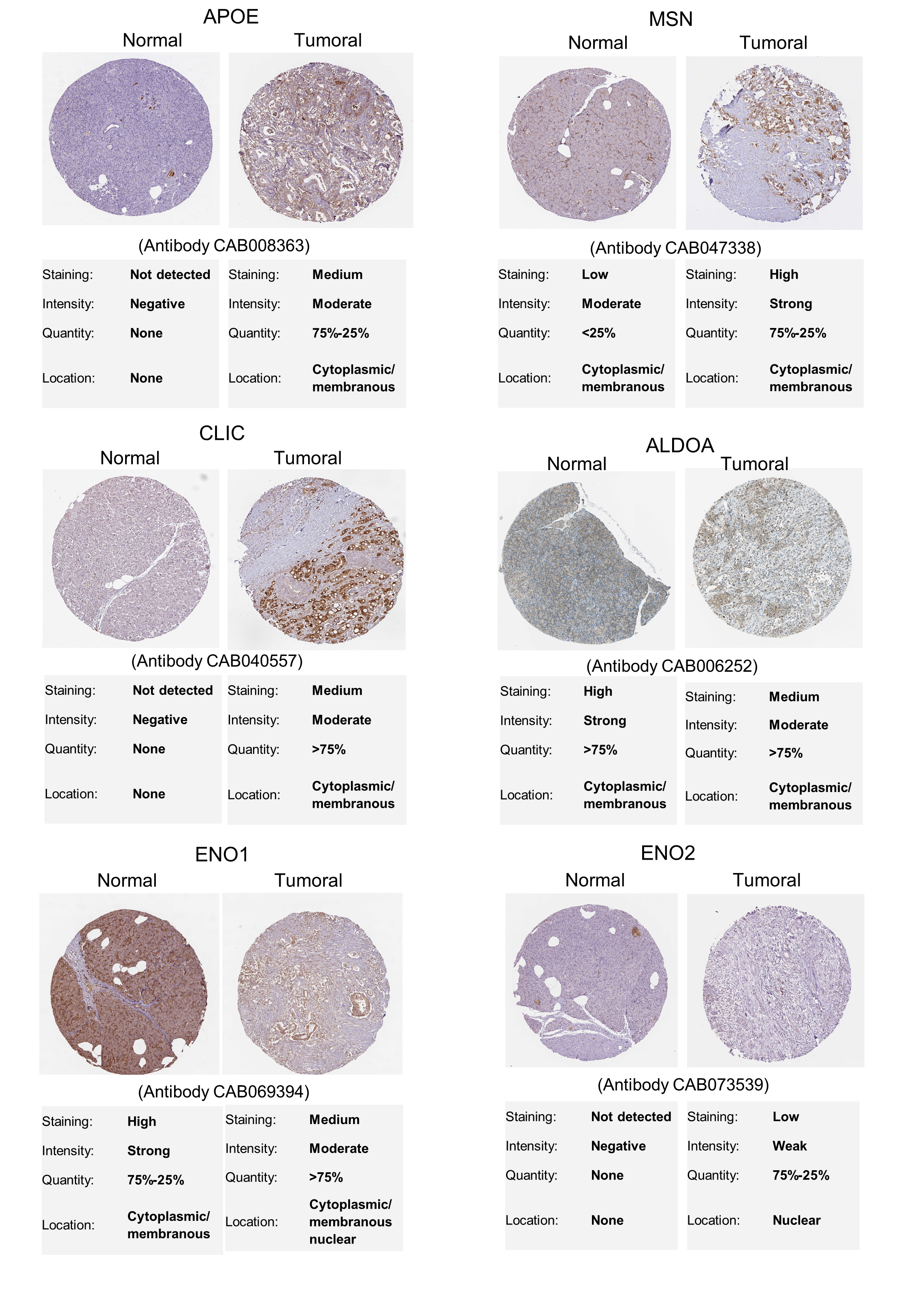

Supplement: Supplementary file 1 [file cancers-12-00716-s001.zip › Supplementary Figure 4 b.TIF]

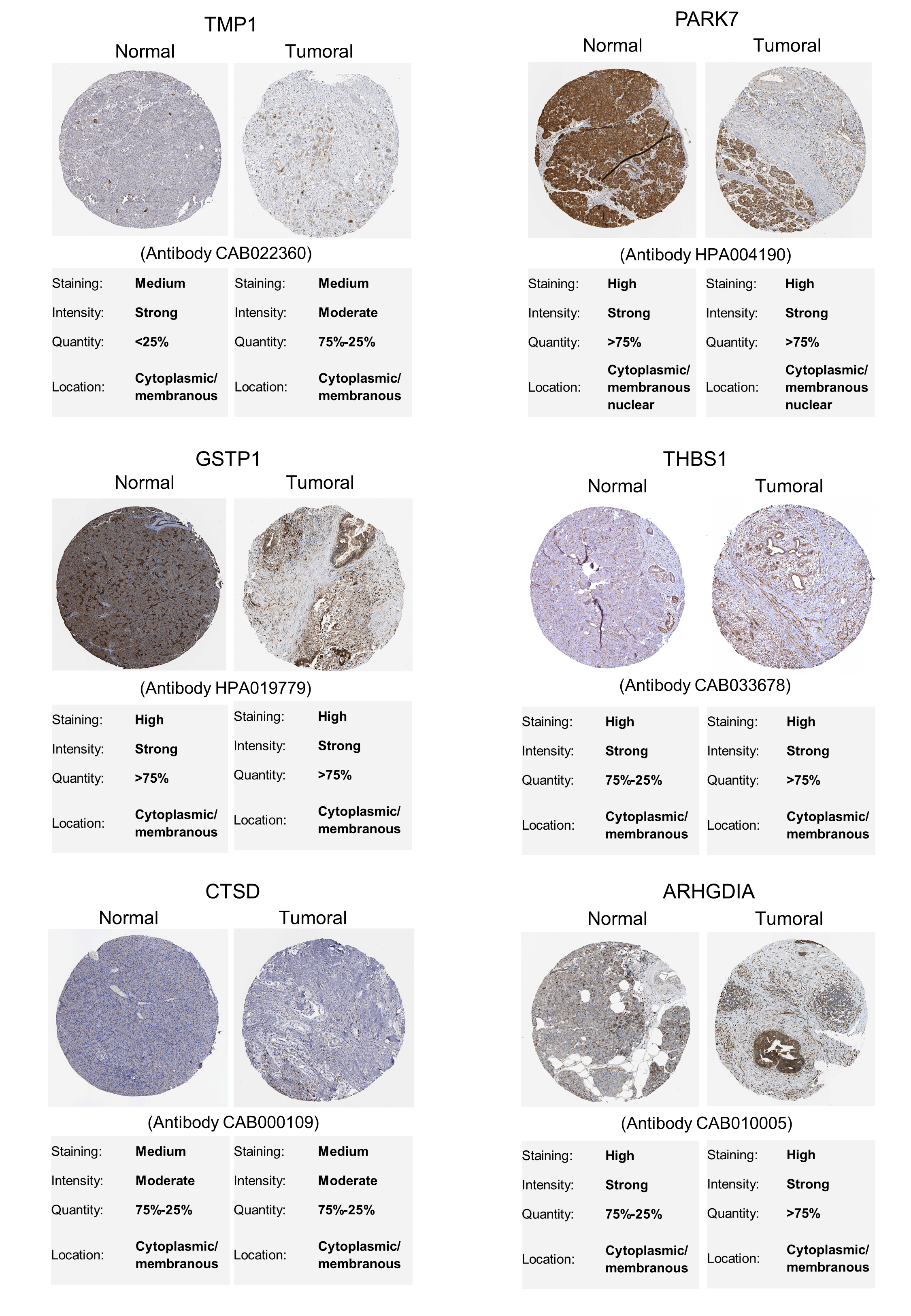

Supplement: Supplementary file 1 [file cancers-12-00716-s001.zip › Supplementary Figure 4 c.TIF]

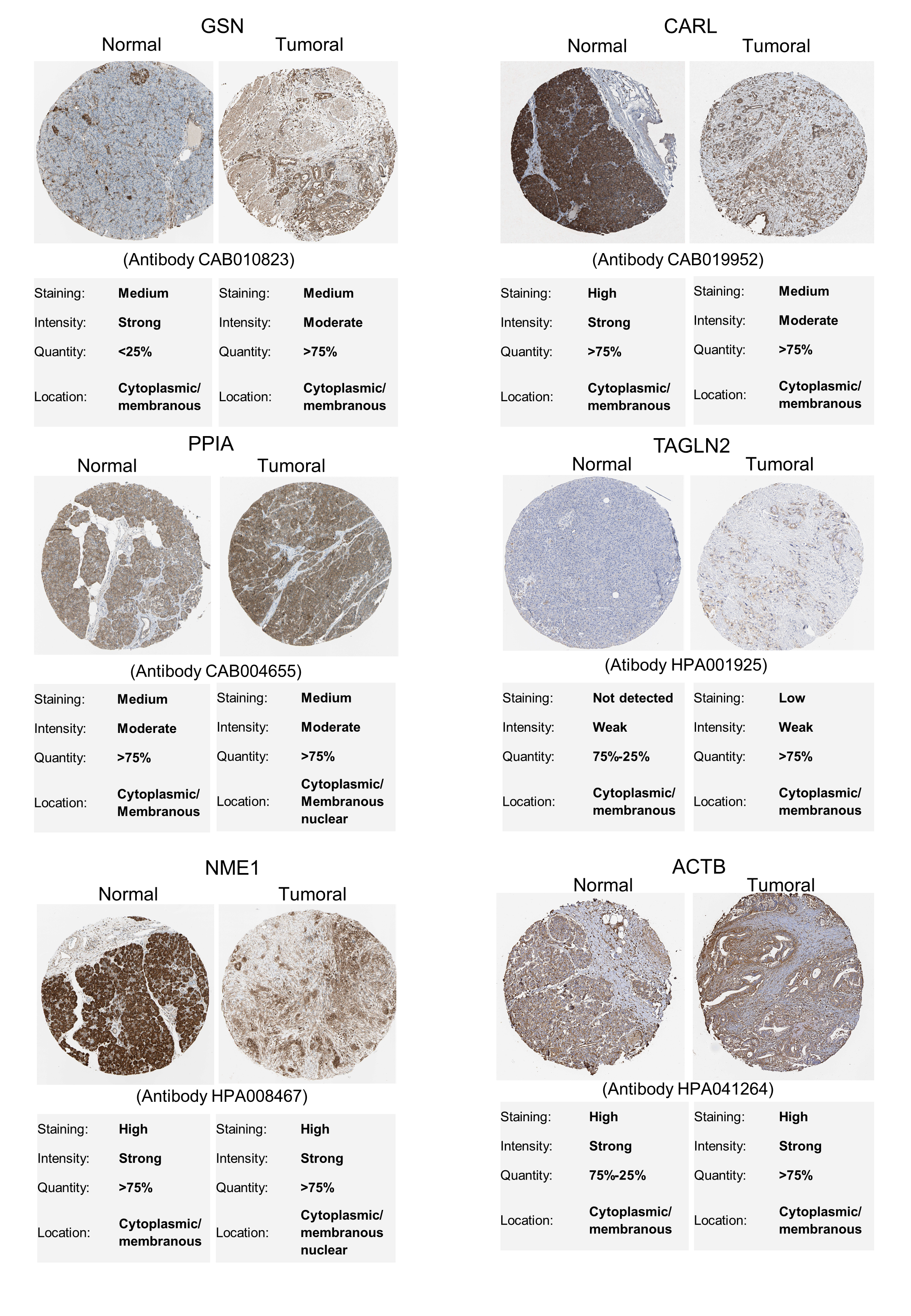

Supplement: Supplementary file 1 [file cancers-12-00716-s001.zip › Supplementary figure 4 d.TIF]

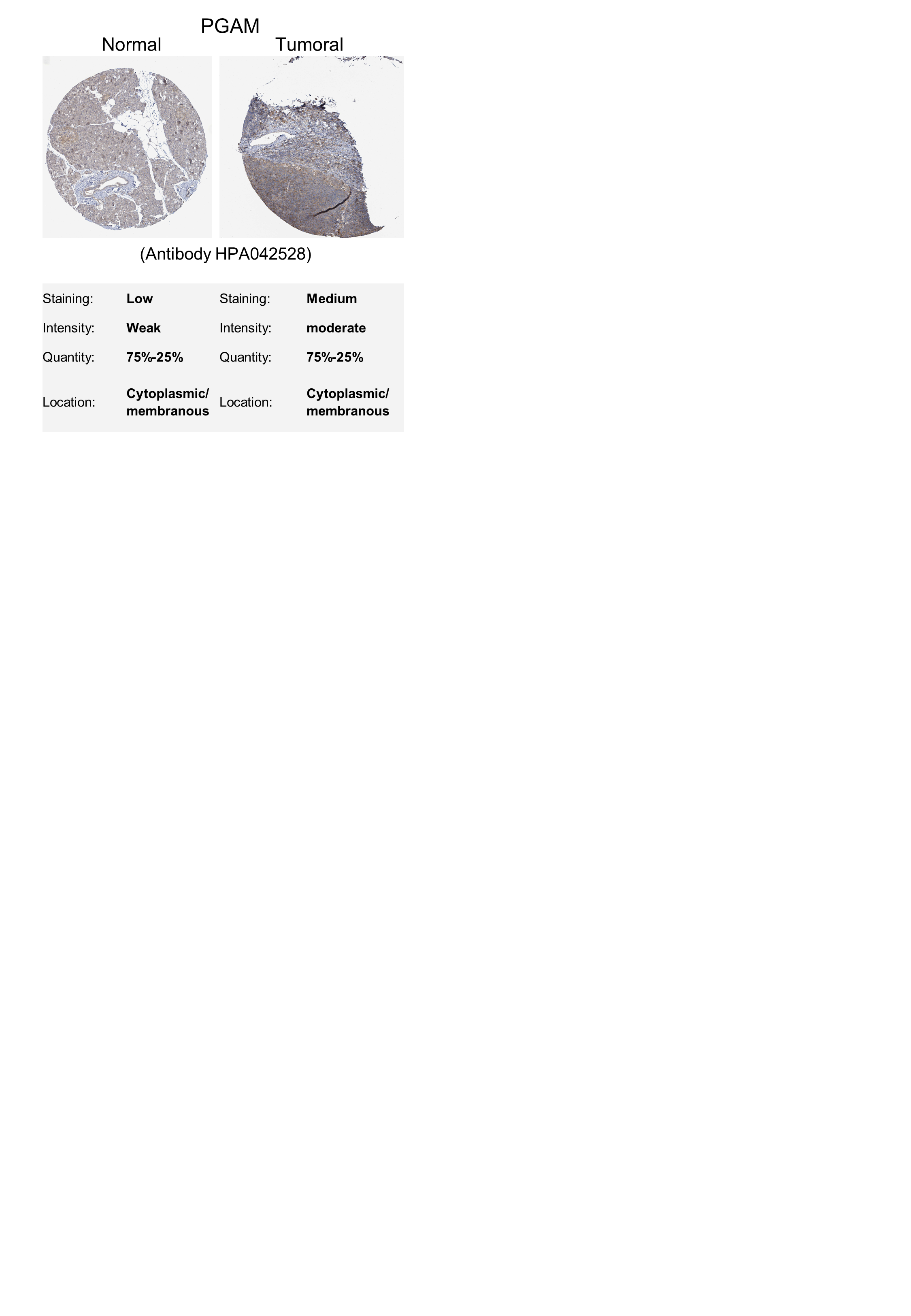

Supplement: Supplementary file 1 [file cancers-12-00716-s001.zip › Supplementary figure 4 e.TIF]
